# Adsorption of Phthalate Acid Esters by Activated Carbon: The Overlooked Role of the Ethanol Content

**DOI:** 10.3390/foods11142114

**Published:** 2022-07-15

**Authors:** Yuanhao Zhou, Bingyu Zhao, Lingxuan Wang, Ting Li, Hong Ye, Shuangyang Li, Mingquan Huang, Xianren Zhang

**Affiliations:** 1 Key Laboratory of Brewing Molecular Engineering of China Light Industry, Beijing Technology and Business University, Beijing 100048, China; zhouyuanhao97@163.com (Y.Z.); zhaoby98@163.com (B.Z.); yqdszhdapxuan@163.com (L.W.); liting120902@163.com (T.L.); lishuangyang@th.btbu.edu.cn (S.L.); huangmq@th.btbu.edu.cn (M.H.); 2 Beijing Laboratory of Food Quality and Safety, Beijing Technology and Business University, Beijing 100048, China; 3 State Key Laboratory of Organic-Inorganic Composites, Beijing University of Chemical Technology, Beijing 100029, China; zhangxr@mail.buct.edu.cn

**Keywords:** activated carbon, adsorption, phthalate acid esters, alcohol–water solutions, application

## Abstract

Ethanol has great effects on the adsorption of phthalate acid esters (PAEs) on activated carbon (AC), which are usually overlooked and hardly studied. This study investigated the overlooked effects of ethanol on the adsorption of PAEs in alcoholic solutions. The adsorption capacities of dibutyl phthalate (DBP) on AC in solutions with ethanol contents of 30, 50, 70, and 100 v% were only 59%, 43%, 19%, and 10% of that (16.39 mg/g) in water, respectively. The ethanol content increase from 50 v% to 100 v% worsened the adsorption performances significantly with the formation of water–ethanol–DBP clusters (decreasing from 13.99 mg/g to 2.34 mg/g). The molecular dynamics simulation showed that the DBP tended to be distributed farther away from the AC when the ethanol content increased from 0 v% to 100 v% (the average distribution distance increased from 5.25 Å to 15.3 Å). The PAEs with shorter chains were more affected by the presence of ethanol than those with longer chains. Taking DBP as an example, the adsorption capacity of AC in ethanol (0.41 mg/g) is only 2.2% of that in water (18.21 mg/g). The application results in actual Baijiu samples showed that the adsorption of PAEs on AC had important effects on the Baijiu flavors.

## 1. Introduction

Phthalate acid esters (PAEs) can improve the molding and processing performance of plastics and can endow materials with good plasticity and tensile strength. Thus, they are widely used as plasticizers in various fields, including construction, food packaging, cosmetics, and in medical equipment. However, PAEs can easily accumulate in humans and have estrogen-like activities, which can lead to several diseases of the reproductive, nervous, and immune systems [1]. In addition, PAEs can be transported over long distances, resulting in their wide distribution in indoor dust, food, water, air, and soil. Therefore, PAEs have become some of the most important emerging contaminants and have attracted significant attention in environmental protection and food safety [2]. In 2012, six kinds of PAEs including dimethyl phthalate (DMP), diethyl phthalate (DEP), n-dibutyl phthalate (DBP), butylbenzyl phthalate (BBP), di(2-ethylhexyl) phthalate (DEHP), and di-n-octyl phthalate (DnOP) were classified as priority controlled contaminants by the US [3]. The China National Environmental Monitoring Center has also designated DMP, DEP, and DnOP as priority pollutants for monitoring [4]. Therefore, the removal of PAEs is of great importance.

Among the reported removal methods, microbial degradation is one of the main technologies, but its effectiveness is limited [5]. Advanced oxidation processes (AOPs) are vulnerable to oxidation conditions and have unstable removal rates, but have high PAE removal efficiencies [6]. However, the treatment of AOP degradation byproducts and the energy consumption of these processes must be considered [7]. Adsorption is the most commonly adopted method for PAE removal due to its simple operation method and low cost [8]. Furthermore, PAE adsorption is a physical reduction technique with low impacts on the other components in the system, allowing plasticizers to be recycled. It is suitable not only for the treatment of polluted water bodies, but also for the removal of harmful substances from foods and beverages with more complex components. The adsorption performance is determined by the adsorbent.

Many studies have focused on the preparation and modification of adsorbents for PAE removal. As early as 1982, Sullivan et al. [9] explored the adsorption of DBP and DEHP on clay. However, instead of clay, activated carbon (AC) has been widely applied in industries for PAE removal due to its excellent adsorption performance for PAEs. For example, commercial peat-based AC achieved an adsorption capacity of 858 mg/g for DEP in water [10]. In addition, AC from *Albizia* *julibrissin* pods obtained an adsorption capacity of 977 mg/g for DBP in water via multiple adsorption interactions, including electrostatic interactions and dipole–dipole, quadrupole, and hydrogen bonding [11]. Recently, biochar (BC), which has a structure similar to that of AC, has been proposed as an alternative to AC because of its much lower cost. However, the adsorption performance of BC is highly dependent on the type of raw material and the pyrolysis conditions, and ranges from approximately 2000 mg/g [12] to 2 mg/g [13] for the adsorption of DMP or DEP from water. Novel (modified) nanomaterials have also been developed for PAE adsorption. Gao et al. [14] modified magnetic Fe_3_O_4_ particles with an amphiphilic structure and achieved good adsorption of PAEs with long chains. The good adsorption performance was due to the hydrophobicity introduced by the amphiphilic structure and the good dispersion of the modified Fe_3_O_4_ particles in the water. Similarly, Diao et al. [15] modified mesoporous silica particles with 3,3,3-trifluoropyl trimethoxypolysilane to increase the adsorption capacity of the material for DBP to more than eight times that of the unmodified adsorbent. In addition to manipulating the hydrophilicity or hydrophobicity of various substrate materials, Khan et al. [16] used ZIF-8 for DEP adsorption from water; however, the adsorption performance of ZIF-8 was only 1/3 that of AC. Yang et al. [17] prepared a molecularly imprinted polymer on the surfaces of magnetic particles using DEHP as a template molecule to remove PAEs from methanol.

There are two issues worth noting in the studies described above. First, most of the adsorption performances investigated were for PAE–water systems, and other environmental systems, such as ethanol solutions, are seldom considered. It is well known that PAEs are much more soluble in ethanol than water. The effect of the ethanol content on PAEs represents the removal law of PAEs in a broad category of alcoholic beverages. As a result, alcoholic systems, such as alcoholic beverages, are more easily contaminated with PAEs than water and deserve significant attention in terms of their environmental protection and food safety. Furthermore, the removal of PAEs in Baijiu is much more difficult than the removal of PAEs in water environment. In addition to the PAE removal, retaining the volatile organic compounds in alcoholic beverages in order to guarantee the original flavor is a very challenging problem. However, there are no reports focusing on the effect of the alcohol content on the adsorption removal of PAEs, which may be an important but often overlooked factor in the removal of this contaminant. Second, the adsorption interactions between PAEs and adsorbents are less discussed or speculated in terms of the adsorbent structure. Although molecular simulations can help to gain insight into the adsorption process at the molecular level, molecular dynamics (MD) simulations describing PAE adsorption have not been reported.

Based on the above, in this study the effects of ethanol on PAE adsorption were systemically investigated. AC and DBP were used as the adsorbent and main target, respectively, because AC is the most widely applied absorbent and DBP is one of the most important PAEs in alcoholic beverages. The kinetics and thermodynamics of the adsorption in aqueous solutions with different ethanol contents were studied. The effects of the temperature and pH on adsorption were also discussed. MD simulations were used to describe the adsorption process of PAEs on AC in various alcohol–water systems. The research design of this paper is shown in Figure S11.

## 2. Materials and Methods

### 2.1. Chemicals and Materials

The commercial AC JT-207 was purchased from Hwrk Chemical Co., Ltd. (Beijing, China). Jiang-flavored Baijiu I (alcohol content 53 v%, Baijiu I) and Jiang-flavored Baijiu II (alcohol content 53 v%, Baijiu II) were purchased from a local store (Beijing, China). The 4-octanol (internal standard) was purchased from Tokyo Chemical Industry Co., Ltd. (Tokyo, Japan). The hydrochloric acid (HCl, chemical purity) was purchased from the Beijing Chemical Plant (China). The sodium hydroxide (NaOH) was purchased from Sinopharm Chemical Reagents Co., Ltd. (Beijing, China). The pyridine was purchased from Mindray Chemical Technology Co., Ltd. (Shanghai, China). The ethanol and methanol were provided by Titan Technology Co., Ltd. (Shanghai, China). The acetic acid and DBP were obtained from Adamas Reagent Co., Ltd. (Shanghai, China). The DMP and dipropyl phthalate (DPP) were supplied by Tokyo Chemical Industry Co., Ltd. (Tokyo, Japan). The DEP was supplied by Maclin Biochemical Technology Co., Ltd. (Shanghai, China). The DEHP and DnOP were purchased from Aladdin Biochemical Technology Co., Ltd. (Shanghai, China). All chemicals were of analytical purity, except hydrochloric acid, which was used as received without further purification.

### 2.2. Characterization

The surface morphologies of the AC samples were characterized using scanning electron microscopy (SEM; EM-30 Plus, COXEM, Daejeon, Korea). For better observation, the samples were sprayed with gold before testing to increase their surface conductivities.

The AC surface functional groups were identified via Fourier transform infrared spectroscopy (FTIR, Thermo Nicolet iS5, Thermo Fisher Scientific, Waltham, MA, USA). The samples were characterized in the infrared region using the potassium bromide sheet method at 25 °C with wavenumbers ranging from 4000 to 600 cm^−1^, 32 scans and 4 cm^−1^ resolution. The sample profiles were retrieved and analyzed using an infrared atlas database.

The nitrogen adsorption–desorption isotherms and other pore properties were determined at a pretreatment temperature of 200 °C and time of 6 h using a surface-area pore analyzer (ASAP 2460, Mac, Atlanta, GA, USA). The Brunauer–Emmett–Teller (BET) method was used to calculate the specific surface area.

### 2.3. Isoelectric Point Measurement

The pH drift test was used to determine the point of zero charge (pH_pzc_) [18]. The pH of the 50 mL ethanol–water solution was adjusted from 3 to 9 using 0.010 mol/L HCl and NaOH. The AC (0.050 g) was added to the solution in a conical flask and stirred at 30 °C for 3 h at 150 rpm. Next, the final pH was measured and plotted against the initial pH. Finally, the pH at which the curve crossed the pH_initial_ = pH_final_ end line was taken as pH_pzc_. Each measurement was repeated at least twice. The pH was measured using a high-accuracy pH meter (accuracy ±0.002 pH, Sanxin Meter Factory, Shanghai, China).

### 2.4. Adsorption Research

Batch adsorption experiments were conducted to test the adsorption performance of the AC [19].

First, various PAE solutions (100 mL each) with different concentrations were prepared using water, ethanol, or ethanol–water mixtures as solvents in glass plug conical flasks (except for the kinetic experiments). Because 2.00 mL of solution was removed and tested at specific time points, the volume of each solution was increased to 200 mL and the amount of AC was increased to 0.100 g for the kinetic experiments. Then, to prevent the volatilization of ethanol during the experiment, the bottle mouth of the flask was covered with a sealing film. Next, AC was added to the PAE solution and dispersed ultrasonically (Kun Shan Ultrasonic Instruments Co., Ltd., Kunshan, China) for 1 min. Finally, the adsorption solution was quickly transferred to a thermostatic water bath vibrator (Beijing Tian Lin Heng Tai Technology Co., Ltd., Beijing, China) and shaken at 150 rpm for 72 h in a dark room at the desired temperature to ensure adsorption equilibrium. In addition, two Baijiu samples (Baijiu I and Baijiu II) from the local market were adopted for the adsorption experiments. Baijiu I was stored in plastic containers containing PAEs for one year; Baijiu II was added with DBP or 5 PAEs, and the final concentration of each PAE was 10 mg/L. At the same time, in order to exclude the possible volatilization loss of volatile compounds, when the adsorption experiment was carried out, a control group was set up under the same conditions but without adding AC.

Except for the isothermal adsorption experiments, which were conducted at 20, 30, and 40 °C, the experiments were conducted at 30 °C. The initial concentration of PAEs in each experiment was 10 mg/L, except that high concentration gradients of 20, 30, 40, 50, and 60 mg/L were used in the isotherm adsorption experiment. In all experiments except for the adsorption experiments of multicomponent PAEs (DMP, DEP, DBP, DEHP, and DnOP), single-component DBP was used as the adsorption study target. All adsorption experiments were repeated at least twice, and each sample was tested at least thrice.

A small quantity of sample was withdrawn from the glass plug conical flask using a syringe at the specified time intervals to monitor changes in the concentration of PAEs in the solution. After filtration through a 0.45 μm PTFE filter membrane, the PAE concentration was determined via gas chromatography–mass spectrometry (GC-MS, Thermo Fisher, America). DPP was used as the internal standard. The ratio of the peak area of the measured substance to the DPP was used to determine the content (detection time and PAE peak shape values are presented in Table S1 and Figure S1). The detailed conditions are described in the Supplementary Materials (Text S1). The same goes for the flavor detection method and the concentration calculation method (Text S2).

The Langmuir, Freundlich, pseudo-first-order kinetics, pseudo-second-order kinetics, intra-particle diffusion kinetics, and Van’t Hoff models were used to investigate the mechanism of DBP adsorption on AC. The detailed models are shown in the Supplementary Materials (Text S3).

### 2.5. Molecular Simulation Calculations

Molecular dynamics (MD) simulations were carried out using the Forcite and Amorphous Cell modules in Materials Studio 7.0 (Accelrys Software Inc., San Diego, CA, USA). The time step was set to 1.0 fs for all dynamics runs. For the AC, the structure was constructed based on a random combination of graphene micro-elements. The all-atom model was adopted for the water, ethanol, and DBP molecules. The AC was placed in the middle of the box along the *z*-axis with two DBP molecules placed on either side of it. The system was then filled with ethanol and water at different concentrations. A periodic structural unit with dimensions of 23 × 23 × 80 Å was used. The density of the AC was approximately 1 g/cm^3^. The initial configuration is shown in Figure S5, in which the COMPASS II force field [20] was used. The Nose algorithm was used to maintain the temperature (303 K) with the Q ratio set to 0.01, and the Berendsen method was adopted to maintain the pressure (0.1 MPa), with a decay constant of 0.1 ps. The electrostatic interaction was computed using the Ewald summation, and the van der Waals interaction was evaluated using the atom-based scheme with a cutoff of 12.5 Å. All of the resulting atomistic structures were first minimized using the smart minimizer method (50,000 steps) to eliminate the local nonequilibrium, then MD runs (NPT, 1000 ps) were performed to further equilibrate the models. Finally, the data were collected for analysis through the NVT ensemble balance for 1000 ps.

### 2.6. Aroma Profile Analysis

A Thermo Fisher Trace GC–MS equipped with a single-quadrupole (ISQ) was used in this study. The instrument operation and analytical methods are detailed in the Supplementary Materials (Text S2). Eight panelists with experience in olfactory experiments were recruited from the Key Laboratory of Brewing Molecular Engineering of China Light Industry. The samples before and after adsorption were simultaneously presented to the sensory panelists to evaluate the sensory differences between the two samples. The panelists were provided with 10.0 mL of the Baijiu samples in glass bottles (20.0 mL) coded with numbers. Evaluation experiments were performed in a sensory control room at 20 ± 1 °C with a humidity of 45–50% [21]. After sniffing a sample, the panelists would rest for a few minutes before sniffing the next sample. Each sample was repeated three times.

## 3. Results and Discussion

### 3.1. PAE Adsorption

#### 3.1.1. pH Influence

The pH of a solution is an important indicator of its properties. The pH affects the type of charge on the AC surface groups as well as the ionization state of the target molecules in the solution, thereby affecting the adsorption. The ionization equilibrium constant (pK_a_) describes the ability of a substance to dissociate hydrogen ions in water. When the pH value of a solution is higher than its pK_a_, the substance is ionized and exists in an ionic state. The pK_a_ values of the two carboxylic acid groups of phthalic acid (H_2_-PA) are 2.9 and 5.4 [22]. Previous studies have shown that PAEs have different ionization effects at different pH values [23]. At low pH, DBP existed as H_2_-PA. As the pH increased, DBP was deprotonated and became negatively charged, as shown in Figure S6.

Unlike in previous studies, the solution pH was adjusted using organic acids and bases such as acetic acid and pyridine instead of HCl and NaOH. Most alcoholic beverages contain predominantly organic acids and bases (typically acetic acid and biogenic amines) in their system. Thus, given their practical applications, organic acids and bases were used to investigate the effects of the pH on the DBP adsorption.

Using the pH drift method, the pH_pzc_ of AC in both water and 50 v% solution was 6.7 (Figure S7). This means that the pH of the solution was less than 6.7 and the AC surface was positively charged; otherwise, it would be negatively charged. The pH_pzc_ of DBP in water is reported to be approximately 2 [24]. The electrostatic force between the AC and DBP changes with the pH and affects the adsorption capacity. Therefore, the effects of the pH change on the adsorption capacity were investigated.

**Figure 1:**
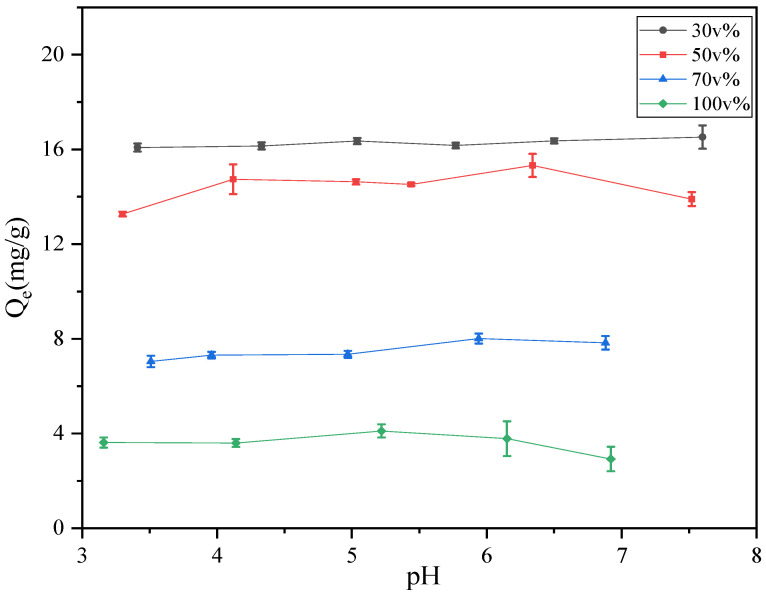
The effect of the solution pH on the adsorption capacity for DBP. Each volume fraction in the legend in this paper is the ethanol content.

As shown in Figure 1, the adsorption capacity did not change significantly with pH values ranging between pH 3 and 8 (even over pH_pzc_), which is quite different from the phenomenon in water systems, where the adsorption performance usually decreases when the pH exceeds pH_pzc_ [25]. The possible explanations are as follows: (1) The adsorption of DBP on AC and is not primarily due to electrostatic interactions in alcohol-containing aqueous solutions. The degree of ionization of weak organic acids and alkalis in alcoholic solutions is not as complete as in water. (2) The main adsorption interaction may be due to hydrophobicity [11,12] along with the hydrogen bonding and π-π EDA, as confirmed by FTIR (Figure S3 and other characterization results for AC, such as the particle size in Figure S2 and pore size data in Figure S4 and Table S2, are detailed in the Supplementary Materials).

This result also shows that for fermentation solution systems that generally contain acetic acid, such as for winemaking, the pH has a negligible effect on the PAE adsorption removal. This means that the AC has a wider range of applications in alcohol-containing systems. Therefore, the pH was not adjusted in subsequent experiments.

#### 3.1.2. Adsorption Kinetics

**Figure 2:**
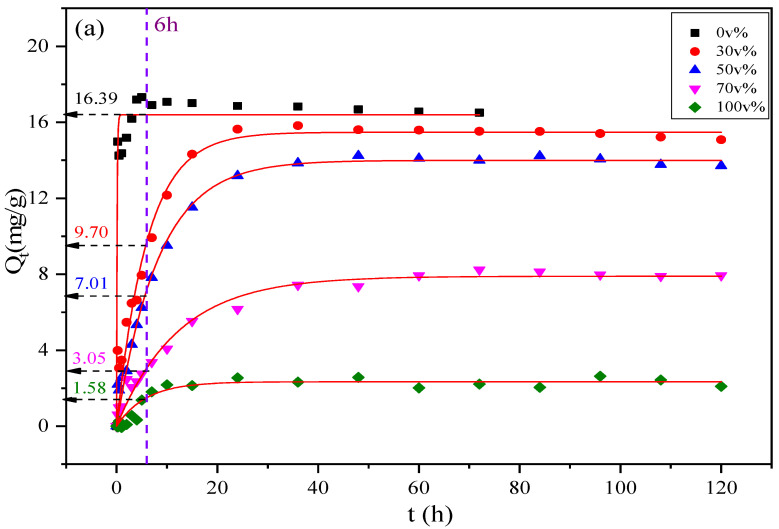

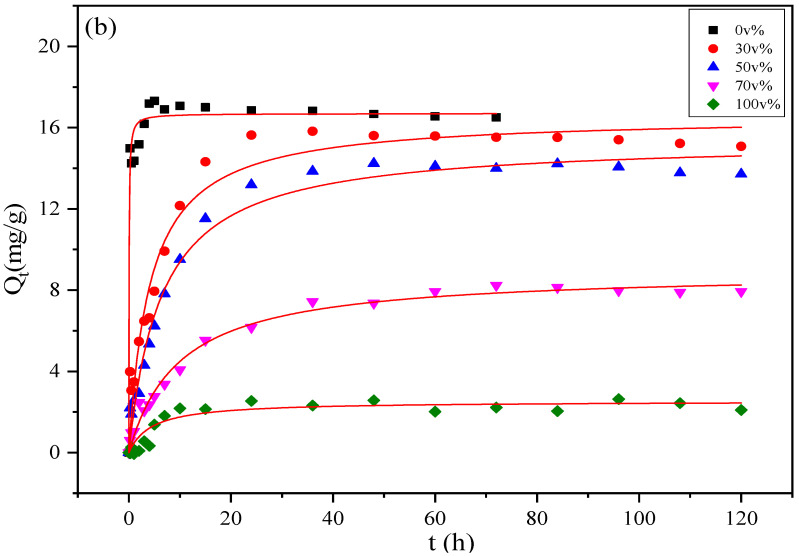
Adsorption kinetic models: (**a**) pseudo-first-order kinetic model; (**b**) pseudo-second-order kinetic model.

Figure 2 shows the pseudo-first-order kinetic model (PFM) and pseudo-second-order kinetic model (PSM) fitting curves for the adsorption of DBP onto AC.

As shown in Figure 2, the adsorption capacity increases as the adsorption time increases. The initial adsorption rate in water was very high. This was because at the beginning of the reaction, some oxygen-containing functional groups and active sites on the AC surface participated in the adsorption and removal of DBP [26]. The large specific surface area of the AC particles allowed the DBP molecules to be quickly adsorbed on the surface and fill the pores, resulting in a rapid increase in the adsorption capacity. When the adsorption experiment was continued for 6 h (Figure 2a), the adsorption of DBP on AC in water reached equilibrium (Q_0v%_ = 16.39 mg/g). However, when the adsorption experiment was conducted in a solution containing 30 v% ethanol for 6 h, the adsorption capacity of AC for DBP was only 53% of that in water (Q_30v%_ = 9.70 mg/g). For the systems with higher ethanol contents, the adsorption became even more difficult. For example, in the 100 v% ethanol solution, the adsorption capacity was only 9% of the adsorption capacity in water (Q_100v%_ = 1.58 mg/g). The presence of ethanol greatly affects the adsorption of DBP on AC.

While the adsorption in water required 6 h to reach equilibrium, in 30, 50, 70, and 100 v% ethanol solutions, the adsorption equilibrium times were approximately 6.7, 8.3, 11.3, and 12 times that in water, respectively. Ethanol has a significant effect on the kinetics of the adsorption of DBP on AC, which outweighs its effect on the equilibrium adsorption capacity. This also means that sufficiently long adsorption times are necessary when AC is used for PAE removal in alcoholic systems. For the following adsorption experiments, 72 h was used as the equilibrium adsorption time to allow the adsorption in each alcohol-containing solution to reach equilibrium.

The following arguments can be used to explain the inhibitory effect of ethanol on PAE adsorption.

First, it can be attributed to the complexation between molecules in the systems. The hydroxyl moiety (-OH) of ethanol can combine with water, and the ethyl moiety (CH_3_CH_2_-) of ethanol can combine with hydrophobic DBP. Therefore, the presence of ethanol effectively improves the solubility of DBP in aqueous solution. This phenomenon is known as the co-solvent effect of ethanol [27]. This combination also retards the diffusion of DBP to the adsorbent.

Second, it can be explained by the Hildebrand solubility parameters of ethanol, water, and DBP. The solubility parameters of DBP, ethanol, and water are 19.2 (MPa)^1/2^ [28], 26.2 (MPa)^1/2^, and 48.0 (MPa)^1/2^ [28], respectively. According to the principle of compatibility, DBP has a higher affinity for ethanol than for water. Therefore, the higher the ethanol content in the solution system, the more DBP tends to distribute in the solution instead of on the adsorbent.

Finally, AC is a type of porous material, and the pore filling effect indicates that the smaller ethanol molecules will diffuse preferentially into the AC over DBP. After the active sites of the AC are occupied, the DBP can enter the AC only through the pores occupied by ethanol to access the inner unoccupied sites. However, the affinity of DBP for ethanol hinders its diffusion into the AC. Therefore, the higher the volume fraction of ethanol, the less likely the DBP will be adsorbed from the solution and the longer it will take for the adsorption to reach equilibrium.

Table S3 lists the relevant kinetic parameters. By comparing the correction fitting coefficient R^2^ (adj) and the similarity between the theoretical and actual equilibrium adsorption capacities of the two kinetic fitting models, it can be concluded that the PFM is suitable for describing the adsorption of DBP on AC in alcohol-containing solutions and the PSM is suitable for describing the adsorption of DBP on AC in water systems. The PSM assumes that the adsorption is primarily a surface chemical adsorption process in which electrons are shared and exchanged between the adsorbent and adsorbate. This also shows that the adsorption of DBP on AC in the water system may be dominated by chemical actions such as hydrogen bonding, which is consistent with previous studies [29]. The PFM may be more suitable for the adsorption of DBP on AC in alcohol-containing systems. The PFM assumes that the adsorption is controlled by the diffusion step and is mainly used to describe monolayer adsorption via boundary diffusion. Therefore, the adsorption of DBP on AC in the alcohol-containing system may be dominated by pore filling after the diffusion.

The analysis of the above kinetic data showed that ethanol greatly affected the diffusion of DBP into AC during adsorption. Therefore, another kinetic model, the intra-particle diffusion model, was used to explain the adsorption process in more depth.

#### 3.1.3. Intra-Particle Diffusion Equation

Figure S8 shows the fitting curve of the intra-particle diffusion model for the adsorption of DBP on AC, and the relevant parameters are listed in Table S4.

After fitting the adsorption data using intra-particle diffusion, a two-step linear relationship was observed, which indicated that the adsorption split into two different stages over time (Figure S8). There was some deviation between the fitted straight line and the origin; therefore, internal diffusion was not the only factor limiting adsorption [30].

Here, k_ip_ is the diffusion rate constant and C is the fitting intercept related to the thickness of boundary layer. The intercept is non-zero in all systems, indicating that intra-particle diffusion is not the only factor controlling the adsorption rate (Table S4); liquid film diffusion may also affect the adsorption.

In stage I of the diffusion, the intercept in the water was 12.32, which was much higher than that in the ethanol-containing systems (from 0.88 to −0.44). This clearly shows that the DBP adsorption in water was not affected by internal diffusion. As the ethanol content increased, the k_ip_ decreased from 3.25 to 0.7 (a decrease of 78.46%). This decrease indicates that the presence of ethanol and the increase in ethanol content greatly affected the internal diffusion rate of the DBP, thereby affecting the adsorption progress.

In stage II of the diffusion, as the ethanol content increased, the ethanol filled the AC pores, reducing the number of adsorption sites. DBP readily forms molecular clusters with ethanol, which hinders the diffusion of DBP in the pores, and the intercepts decreased with increasing ethanol content. This is consistent with the previous adsorption kinetics model, where the adsorption capacity dropped suddenly when the ethanol content exceeded 50%.

Given the conclusions drawn from the above data, we further investigated the effect of temperature on DBP adsorption in alcohol-containing solutions.

#### 3.1.4. Adsorption Isotherms

**Figure 3:**
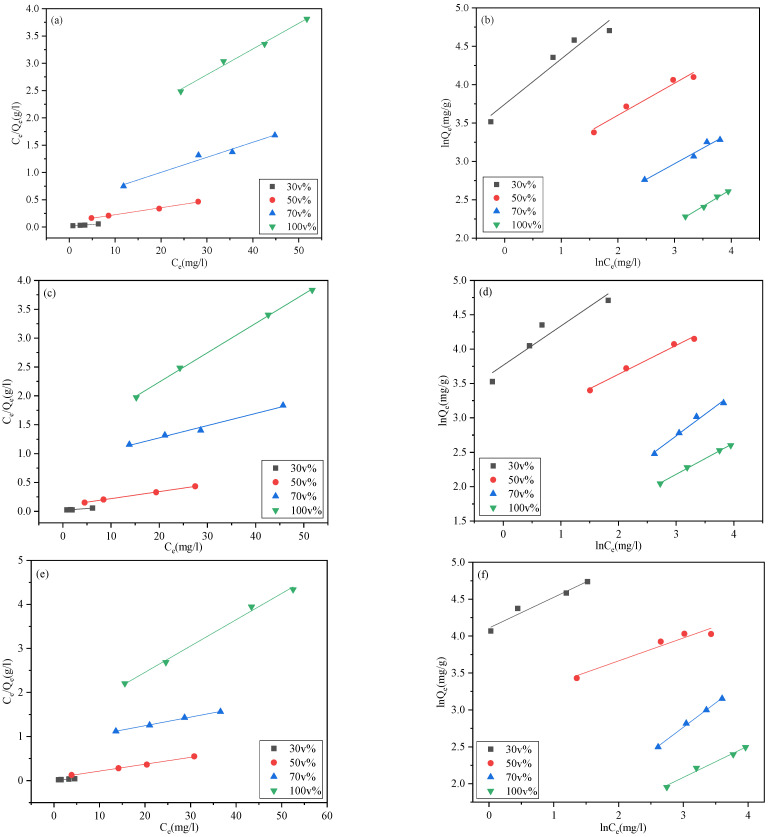
Adsorption isotherm models: (**a**,**c**,**e**) 20 °C, 30 °C, and 40 °C Langmuir adsorption isotherm equations; (**b**,**d**,**f**) 20 °C, 30 °C, and 40 °C Freundlich adsorption isotherm equations.

Experiments were conducted at 20, 30, and 40 °C to investigate the influence of temperature on DBP adsorption. The Langmuir and Freundlich adsorption models were selected to fit the experimental data. Figure 3 shows the fitting curves of the two adsorption isotherm equations for the adsorption of DBP on AC. The relevant parameters are presented in Table S5.

The Langmuir model (0.96 < R^2^ < 0.99) seems to fit slightly better for the isotherm data than the Freundlich model (0.90 < R^2^ < 0.99) (Table S5). These results indicate that the adsorption of DBP on AC is not only a homogeneous adsorption process, but also involves a large portion of heterogeneous sorption behaviors [19]. In general, as ethanol content increased, the equilibrium coefficients K_L_ and K_F_ (reflecting the adsorption affinities of DBP for AC) gradually decreased, and Q_m_ decreased by 87–90%.

**Figure 4:**
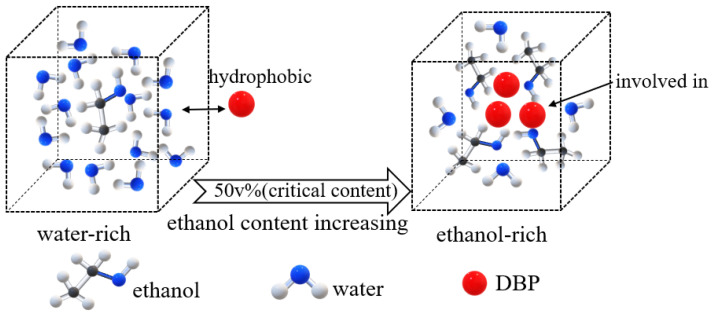
Mechanism diagram of DBP and water–ethanol clusters.

It is worth noting that parameters such as K_L_ in Table S5 decreased sharply when the ethanol content exceeded 50 v%. It can be seen that at 40 °C, as the ethanol content increased from 30 to 50 v%, the value of K_L_ dropped by approximately 60% (from 0.65 to 0.26) and K_L_ decreased by approximately 92% (from 0.26 to 0.02) as the ethanol content increased from 50 to 70 v%. A similar phenomenon can also be observed from the results presented in Figure 4. The experimental equilibrium absorption capacity did not significantly decrease (about 9.7%) from 30 to 50 v%, but showed a significant decrease (42.7%) from 30 to 70 v% (Table S12). The ethanol volume content of 50 v% seemed to be a demarcation point, after which the adsorption performance of AC for DBP markedly decreased, which has seldom been reported in other studies. This phenomenon can be explained by the arrangement of the molecules in the ethanol–water systems.

In an ethanol–water system, the water and ethanol exist in molecular clusters due to hydrogen bonding. The water molecule acts as an electron donor, and the -OH group in the ethanol serves as an electron acceptor. Furthermore, the -CH- in the ethanol can bond with the oxygen atoms in the water [31,32]; thus, ethanol–water clusters are formed.

In the water-rich systems, most of the ethanol molecules are hydrated by and form clusters with water molecules [33]. This means that the amount of free ethanol is limited, resulting in the limited competitive adsorption of ethanol on DBP. Furthermore, water clusters containing few ethanol molecules also weakly hinder the adsorption of hydrophobic DBP, as shown in Figure 4.

However, ethanol clusters are formed in ethanol-rich systems. The flexible ethanol clusters easily collide with other molecules, causing external energy loss [34]. As a result, ethanol can form clusters with DBP due to its high affinity for DBP, and their collision can adversely affect the DBP diffusion. In addition, free ethanol can form strong competitive adsorption on DBP. The effect of ethanol on the adsorption of DBP is shown in Figure 4.

In this study, 50 v% was determined to be the critical ethanol content. Liu et al. [35] reported that a new ethanol–water cluster can form in aqueous solution with an ethanol content just under 60 v%, and the molar ratio of ethanol to water was calculated to be 1:2. Unlike in the study conducted by Liu, where 60 v% was the critical ethanol content, the threshold in our study was 50 v%. It is speculated that the phenomenon observed in our study is relevant to this reported cluster. Additional studies are required to further this investigation.

In Table S5, the value of n in the Freundlich isotherm adsorption equation reflects the adsorption capacity of AC for DBP. When *n* < 0.5 and 0.5 < *n* <l, adsorption is difficult. However, when *n* > l, the adsorption performance is better. For all ethanol–water systems, *n* > 1, indicating that the adsorption of DBP on AC is a relatively easy process. However, the feasibility of adsorption needs to be further explained by thermodynamic parameters.

#### 3.1.5. Adsorption Thermodynamics

The adsorption behavior at different temperatures is needed to clarify changes in the adsorption energy of the system and the feasibility of the reaction. The Van’t Hoff fitting equation (Text S3) was used to fit the thermodynamic results, and the parameters are presented in Table S6.

Here, ΔG represents the driving force of the adsorption process. As shown in Table S6, when the ethanol content is 30 or 50 v%, the ΔG values are negative, indicating that the adsorption of DBP on AC was a spontaneous process and that the reaction was feasible from a thermodynamic standpoint. When the ethanol content exceeded 50 v%, however, ΔG became positive, indicating that adsorption was very difficult and could not proceed spontaneously. This result is consistent with the sudden drop in equilibrium adsorption capacity at ethanol contents over 50 v%, as shown in Figure 2.

It is well known that the heat of adsorption for chemical adsorption is greater than that for physical adsorption. When the adsorption process is physical adsorption, −20 < ΔH < 40 kJ/mol; however, when the adsorption process is chemical adsorption, −400 < ΔH < −20 or 40 < ΔH < 80 kJ/mol [36]. From the specific ΔH of the experiment, the adsorption process may be primarily physical adsorption. The positive ΔH value indicated that the adsorption of DBP on AC in the alcohol-containing systems was endothermic. The positive ΔS value indicates that the adsorption process causes the chaos in the system to increase.

The experiment described above was conducted using only DBP instead of multicomponent PAEs. Given that multiple PAE contaminations are possible in ethanol–water systems, the adsorption performances of five types of PAEs on AC were subsequently investigated.

#### 3.1.6. Adsorption of Multicomponent PAEs

In our previous study, PAEs were classified into three types according to their solubility in water [8]. DMP and DEP are less hydrophobic and more soluble in water than DBP, and are classified as type I PAEs. However, DEHP and DnOP have longer chains and are less soluble than DBP, and are classified as type II PAEs. DBP is an intermediate type between type I and type II PAEs. According to the solubility parameters, all three types of PAEs dissolve more easily in ethanol than in water (Table S7) and the detailed adsorption data are shown in Table S13.

**Figure 5:**
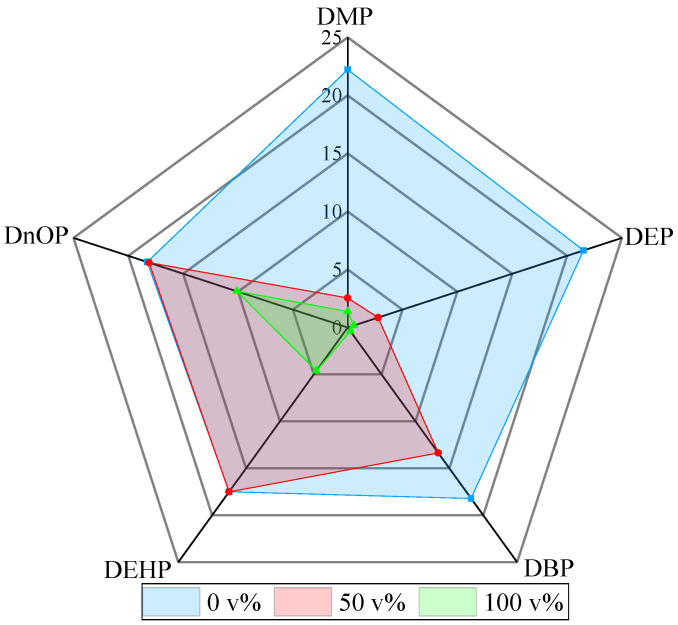
Equilibrium adsorption capacity values of five PAEs in systems with different ethanol contents.

As shown in Figure 5, the adsorption capacities of type I PAEs (DMP and DEP) decreased much more significantly (an approximately 89−94% reduction for DMP at 50 and 100 v% ethanol content) in ethanol–water systems than DBP and type II PAEs (DEHP and DnOP). The main mechanism of adsorption was pore filling for the smaller PAEs [37,38]. Thus, when ethanol occupied the channels of the AC, the narrowed path had a greater influence on the adsorption than the other PAEs. In addition, type I PAEs, which are more soluble in water, more readily formed clusters with the water and ethanol (as shown in Figure 4) than other PAEs, regardless of the ethanol content. However, the more hydrophobic type II PAEs showed only an obvious decrease in adsorption capacity in pure ethanol based on the hydrophobicity adsorption mechanism [38,39].

The unbranched DnOP and branched DEHP are structural isomers. DEHP and DnOP have cross-sectional areas of 0.87 nm^2^ and 0.78 nm^2^, respectively (Table S8). Therefore, the cross-section of the branched molecule was larger, which made the diffusion more difficult, especially when the channels of AC were occupied by ethanol in the 100 v% ethanol system. Thus, compared to DnOP, the adsorption of DEHP was more affected by the increase in ethanol content.

To more accurately and visually describe the inhibitory effect of ethanol on DBP or multicomponent PAE adsorption on AC, MD simulations were used to calculate the changes in the interaction energy levels of AC, PAEs, and the three components of alcohol-containing solutions.

### 3.2. Molecular Dynamics Simulation Analysis

**Figure 6:**
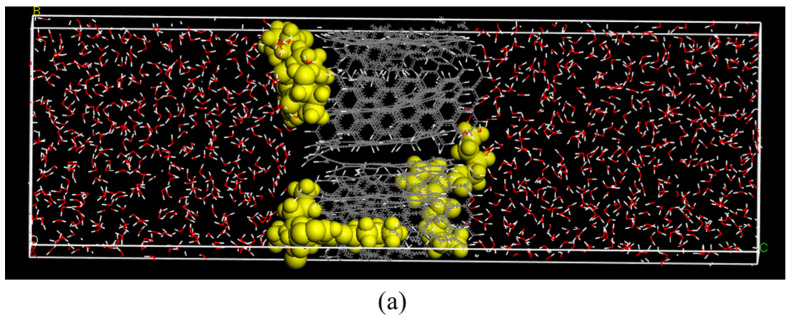

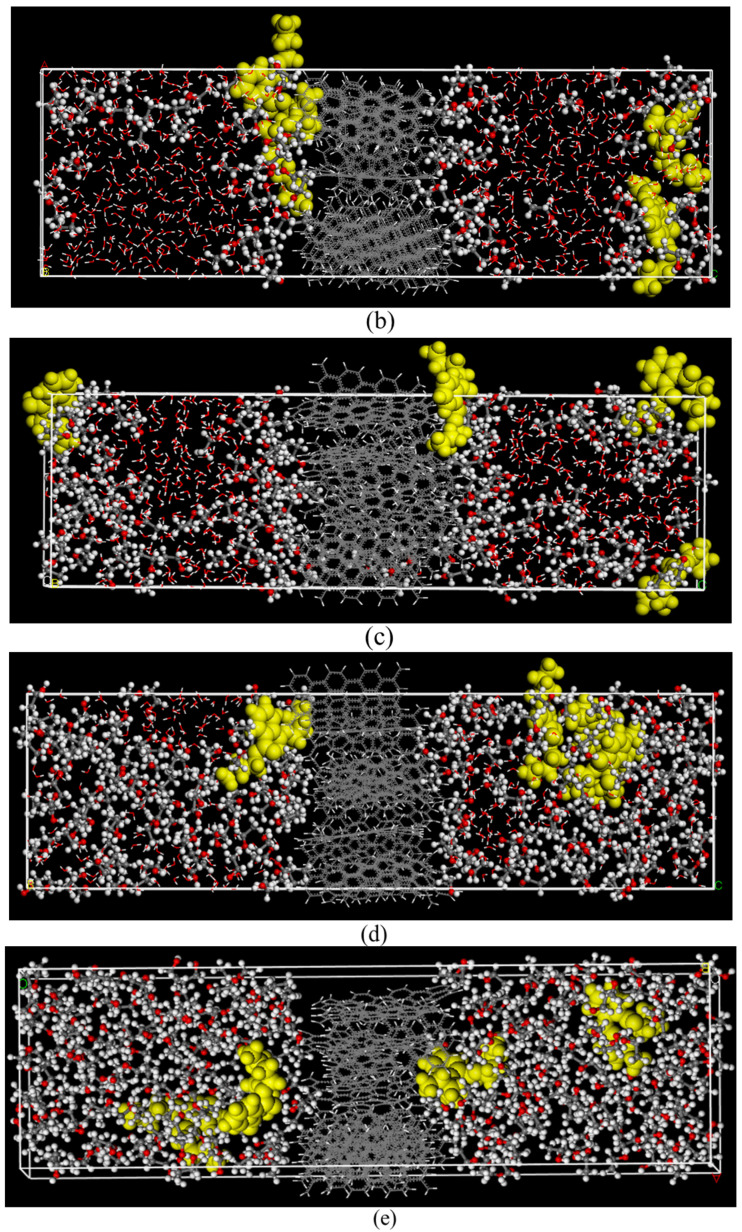
Model snapshots of solutions with different ethanol contents: (**a**) 0 v%; (**b**) 30 v%; (**c**) 50 v%; (**d**) 70 v%; (**e**) 100 v%.

Five adsorption models were established to analyze the distribution of DBP at different ethanol contents. Figure 6 shows snapshots of the models after equilibration. As shown in Figure 6a–e, DBP gradually moves away from AC as the ethanol content increases. This means that ethanol reduces the adsorption effect of AC on DBP. Figure 6b–e shows that the DBP is always surrounded by ethanol, which proves the co-solvent effect mentioned above.

The radial distribution function (RDF) was analyzed to study the effects of the solution environments with different ethanol contents on the DBP adsorption. The expression is as follows:

(1) $$g_{A-B}\left(r\right)=\frac{1}{4{\pi \rho }_{B}r^{2}}\cdot \frac{{\;\mathrm{dN}}_{A-B}}{\mathrm{dr}}$$

where ρ_B_ is the number density of the B particles in the box, and N_A − B_ is the number of B particles around the A particles in the range of r to (r + dr). The relative probability of two groups of atoms or molecules appearing at different distances is represented by RDF. The weak, moderate, and strong interactions between the particles can be explained by comparing the different RDFs [40].

Figure S9 shows the RDF plots for the interactions between different particles. The RDF value is almost zero when r < 2.0 Å. This means that there is no direct interaction between the DBP and the solution or between the DBP and AC [41]. Therefore, the adsorption process is mainly physical adsorption. When 2.6 Å < r < 3.1 Å, there are no obvious peaks in the RDF curves in Figure S9, indicating that no hydrogen bonds exist between the DBP and the solution or between the DBP and AC. As shown in Figure S9a, when the DBP was adsorbed on AC in water, the RDF value between the DBP and AC was much higher than in the alcoholic systems. The RDF value decreased as the ethanol content increased from 0 to 70 v% and then slightly increased at 100 v%. This was because the DBP was better dispersed in the pure ethanol than in water and the DBP moved more freely. Thus, the DBP was more likely to approach the AC in the 100 v% ethanol solution than the 70 v% ethanol solution. In Figure S9b, when r < 5 Å, the RDF value increases with the increasing ethanol content, reflecting the increasing likelihood of DBP molecules to approach the solution. These results are consistent with the above experiment.

To gain more insight into the interactions, the interaction energy of each component in the different solutions was calculated. According to the thermodynamic theory, the interaction energy can be calculated using the following equations [20]:

ΔE_PAEs-AC_ = E_PAEs-AC_ − E_PAEs_ − E_AC_ (2)

ΔE_PAEs-Solution_ = E_PAEs-Solution_ − E_PAEs_ − E_solution_ (3)

where E_PAEs-AC_ is the total energy of the PAEs and AC; E_PAEs-Solution_ is the total energy of the PAEs and solution; E_PAEs_, E_AC_, and E_solution_ are the energy values of the PAEs, AC, and solution, respectively. A positive interaction energy indicates that there is a repulsive force between the two components. However, a negative value indicates attraction. The more negative the interaction energy, the stronger the attraction. The non-bond energy (E_Non-bond_) is mainly composed of the van der Waals energy (E_van der Waals_) and electrostatic energy (E_electrostatic_). The results are presented in Table S9.

As shown in Table S9, in this adsorption system, the van der Waals interactions are dominant and vary greatly, while the electrostatic interactions are secondary, with little variation. This is consistent with the slight changes in the adsorption of DBP on AC at different pH values (see Figure 1). As the ethanol content increased, the △E_Non-bond_ of the DBP–AC became less negative, indicating that the interaction between DBP and AC weakened. However, the △E_Non-bond_ of DBP-Solution became more negative, suggesting that the interaction between DBP and the solution strengthened. When the ethanol content increased from 70 to 100 v%, the interaction between the DBP and AC increased. This is consistent with the results of the RDF analysis. As shown in Table S9, as the ethanol content increases, there is a gradual decrease in △E_van der Waals_ of the DBP, and the solution also indicates the tendency of DBP to gradually approach the solution. The △E_van der Waals_ increased between the DBP and AC, which is disadvantageous for adsorption. This agrees with the conclusions of our previous experiment.

Twenty DBP molecules were added on both sides of the AC in the box to determine the DBP distributions in solutions with different ethanol contents (0, 50, and 100 v%). The concentration distribution of DBP along the z axis is shown in Figure S10. The relative concentration is the ratio of the actual concentration to the average concentration at a certain location. In Figure S10, as the ethanol content increases, the distance of the peaks closest to AC (denoted by the red dots in Figure S10) on both sides increases, while the height gradually decreases (the detail value is given in Table S10). Figure S10a shows that almost all DBP molecules were close to the AC at 0 v% ethanol content, but at 50 v% ethanol content (Figure S10b) some of the DBP molecules were far from the AC. This was due to the interaction between the ethanol and DBP, resulting in the adsorption effect being suppressed. In Figure S10c, the DBP tends to be distributed in the zone far from the AC rather than near it. In general, as shown in Figure S10a–c, the DBP gradually moves away from the AC and the adsorption of the DBP on the AC weakens as the ethanol content increases.

Through simulating the change in ethanol content in the adsorption system and the changes in interaction energy at the molecular level, the general rule for PAE removal in the alcohol-containing system was revealed. In actual systems, such as alcoholic beverages, the presence of volatile compounds also has a certain impact on the removal of PAEs. Therefore, the effect of volatile compounds on the adsorption of PAEs on AC in actual Baijiu was further investigated.

### 3.3. Effects of AC Adsorption on Volatile Compounds

Through the detection and analysis of two kinds of Jiang-flavored Baijiu samples, the main volatile compounds can be divided into three categories, esters, alcohols, and acids. In Baijiu I, the esters, alcohols, and acids accounted for 48.92%, 18.72%, and 19.78%, respectively, while other compounds such as aldehydes and ketones only accounted for 6.59% and 3.21%, respectively. In Baijiu II, the esters, alcohols, and acids accounted for 64.34%, 6.59%, and 28.03%, respectively, while the other compounds such as aldehydes and ketones accounted for 0.68% and 0.29%, respectively.

In Baijiu I, the concentrations of DMP and DBP were 7.12 mg/L and 8.41 mg/L, respectively, and the adsorption capacities of AC for them were 1.57 mg/g and 2.77 mg/g, respectively. Compared with the previous adsorption capacity in the simulated ethanol–water environment (2.55 mg/g for DMP and 13.36 mg/g for DBP; see Section 3.1.6), there was a significant decrease, especially for DBP (decreased by 79.27%). In Baijiu II, the concentrations of the single-component DBP and each PAE in the multicomponent PAEs were added at 10 mg/L, and the adsorption capacity of the AC for the PAEs is shown in Table S11.

It can be seen from Table S11 that the adsorption capacity of the AC for the single-component DBP decreased by 52.39% in Baijiu II, compared with the simulated ethanol–water environment. For the adsorption of multicomponents, it can be clearly observed that the adsorption capacity levels of DMP and DEP are only slightly influenced. However, some volatile compounds (such as ethyl phenylacetate) in Baijiu II have similar structures to DBP, DEHP, and DnOP (especially DBP), and so they affect the adsorption of the three PAEs by the AC. This resulted in a significant decrease in the adsorption capacity levels for the three PAEs, and their adsorption capacity levels dropped to 27.84% (DBP), 48.40% (DEHP), and 61.23% (DnOP) of the original. Among the only Chinese journals, the equilibrium adsorption capacity calculated according to the optimal adsorption conditions reported in the article, our results are relatively better than those from previous studies (Table S14).

In terms of the content changes for the volatile compounds, the esters, alcohols, and acids decreased by 21.99%, 23.91%, and 11.08% in Baijiu I, respectively. At the same time, compared with the control group, the esters pentanoic acid ethyl ester, ethyl L-lactate, dodecanoic acid ethyl ester, and ethyl oleate decreased significantly by 40.22%, 41.09%, 100%, and 100%, respectively; the alcohols 2-phenylethanol, isobutanol, 2-methyl-1-butanol, and 1-pentanol decreased significantly by 15.62%, 25.98%, 100%, and 100% respectively; the acids acetic acid, propionic acid, butyric acid, and caproic acid decreased significantly by 10.35%, 12.86%, 18.95%, and 31.64%, respectively (Table 1). For Baijiu II, the esters, alcohols, and acids decreased by 0.77%, 8.01%, and 5%, respectively, when adsorbing the single-component DBP; when adsorbing multicomponent PAEs, the esters, alcohols, and acids were reduced by 0.42%, 0.41%, and 0.48%, respectively. The ethyl phenylacetate, ethyl palmitate, propionic acid, butyric acid, and caproic acid decreased significantly compared with the other volatile compounds (Table 2).

The total amount of volatile compounds in Baijiu I was 1560.60 mg/L, which decreased by 278.96 mg/L after the adsorption experiment. The adsorption capacity of the AC for DBP decreased by 79.27%. The total amount of volatile compounds in Baijiu II was 894.20 mg/L; after the single-component DBP and multicomponent PAE adsorption experiments, the reductions were 23.09 mg/L and 5.42 mg/L, respectively. In the DBP-containing system, the adsorption capacity of the activated carbon for DBP decreased by 47.61%. In the multicomponent PAE-containing system, the adsorption capacity of the AC for DBP, DEHP, and DnOP decreased by 72.15%, 51.60%, and 38.76%, respectively. This implies that the higher the concentration of volatile compounds in the real Baijiu samples, the less the PAEs were adsorbed. In general, not only ethanol with a high content of 50 v%, but also volatile compounds with relative lower contents of less than 2 g/L have quite great influences on the adsorption of PAEs on AC.

At the same time, a simple olfactory distinction was performed between the original samples and the corresponding samples after the adsorption. All eight panelists indicated that there was a significant difference in their aromas between the samples before and after adsorption. The flavor profiles of the two kinds of Baijiu had changed. In Baijiu I after adsorption, the cellar flavor was weakened, while the fruity flavor was significantly increased. The alcoholic aroma in the adsorbed Baijiu II was more irritating, and the Jiang flavor was stronger after some substances were adsorbed. This implied that the adsorption of PAEs has an important influence on the flavor of Baijiu, because the contents of the aroma compounds in the Baijiu samples were changed.

The above results mean that the removal of PAEs in Baijiu is much more difficult than the removal of PAEs in water environments. In addition to the PAE removal, retaining the volatile organic compounds in Baijiu in order to guarantee the original flavor is a very challenging problem. More work is needed to further this research.

## 4. Conclusions

In this study, the ethanol content was shown to have significant effects on the adsorption of PAEs on commercial AC, which is the most commonly used adsorbent for PAEs. Compared to the PAE adsorption in absolute water, the pH did not affect the PAE adsorption in an alcoholic environment. However, the presence of ethanol resulted in significant reductions in adsorption capacity and an increase in equilibration time. With the increase in ethanol concentration, the ability of AC to adsorb PAE decreases rapidly and the equilibrium time is greatly prolonged. The demarcation point was 50 v%, above which the adsorption performance of AC for DBP worsened significantly. This may be due to the formation of water–ethanol–DBP clusters. The DMP and DEP, which had shorter chains, were more significantly affected by the presence of ethanol than the DEHP and DnOP, which had longer chains, and were only adversely affected when the ethanol content exceeded 50 v%. In the microscopic molecular distribution map of the molecular dynamics simulation, the DBP tended to be distributed further away from the AC with a higher ethanol content, which was consistent with the experimental results. This study shows the considerable effects of alcohol on the adsorption of PAEs on AC and advances the understanding of the PAE adsorption mechanism in alcoholic systems. This is beneficial for the practical application of AC in the removal of PAEs from alcoholic solutions, which are more susceptible to PAE contamination. The application results in actual Baijiu samples implied that the adsorption of PAEs has an important influence on the flavor of Baijiu due to the content change of the aroma compounds in Baijiu. Retaining the volatile organic compounds in Baijiu in order to guarantee the original flavor is a very challenging problem, which requires further research.

## Figures and Tables

**Table 1:** Changes in volatile compounds before and after Baijiu I adsorption.

	CAS	Volatile Compounds	Original Concentration (mg/L)	Concentration after Adsorption (mg/L)	Loss Percentage ^1^ (%)	Concentration of Control Group (mg/L)	Loss Percentage ^2^ (%)
Esters	109-94-4	Ethyl formate	4.143 ± 0.225	4.032 ± 1.705	2.68	4.199 ± 0.094	−1.36
	141-78-6	Ethyl acetate	369.357 ± 72.729	359.33 ± 66.463	2.71	374.109 ± 12.083	−1.29
	108-64-5	Ethyl 3-Methylbutanoate	24.055 ± 0.887	24.868 ± 5.087	−3.38	23.796 ± 5.216	1.08
	539-82-2	Pentanoic acid ethyl ester	4.418 ± 0.571	2.641 ± 2.35	40.22	4.274 ± 0.447	3.26
	123-66-0	Ethyl hexanoate	5.186 ± 0.408	5.579 ± 0.745	−7.58	4.844 ± 0.26	6.60
	106-30-9	Ethyl heptanoate	0.182 ± 0.03 ^ab^	0.146 ± 0.063 ^a^	19.61	0.23 ± 0.005 ^b^	−26.64
	687-47-8	Ethyl *L*-lactate	300.79 ± 13.458	177.208 ± 153.544	41.09	337.414 ± 6.502	−12.18
	106-32-1	Ethyl octanoate	0.985 ± 0.164	1.066 ± 0.167	−8.27	0.968 ± 0.192	1.66
	2441-06-7	Ethyl 2-hydroxy-3-methylbutanoate	0.865 ± 0.047	0.718 ± 0.111	16.98	0.9 ± 0.348	−4.09
	13529-27-6	2-Furaldehyde diethyl acetal	0.75 ± 0.135	0.831 ± 0.133	−10.79	0.933 ± 0.112	−24.39
	10348-47-7	Ethyl 2-hydroxy-4-Methylpentanoate	4.806 ± 0.153	4.727 ± 0.094	1.64	4.621 ± 0.1	3.84
	110-38-3	Ethyl decanoate	0.814 ± 0.15	0.625 ± 0.176	23.26	0.897 ± 0.438	−10.18
	123-25-1	Diethyl butanedioate	2.323 ± 0.409	2.012 ± 0.227	13.39	2.394 ± 0.429	−3.05
	101-97-3	Ethyl 2-phenylacetate	2.747 ± 0.133	2.388 ± 0.101	13.08	2.469 ± 0.952	10.14
	106-33-2	Dodecanoic acid ethyl ester	0.448 ± 0.081	ND ^3^	100.00	0.327 ± 0.06	26.93
	2021-28-5	Ethyl 3-phenylpropanoate	0.235 ± 0.02 ^ab^	0.214 ± 0.032 ^a^	8.77	0.28 ± 0.005 ^b^	−19.36
	124-06-1	Ethyl tetradecanoate	0.792 ± 0.142 ^a^	0.563 ± 0.016 ^b^	28.94	0.72 ± 0.004 ^ab^	9.13
	628-97-7	Ethyl hexadecanoate	11.186 ± 2.76 ^a^	5.922 ± 1.436 ^b^	47.06	6.458 ± 2.78 ^ab^	42.27
	111-62-6	Ethyl oleate	8.606 ± 5.273	ND	100.00	8.599 ± 0.015	0.09
	544-35-4	9,12-Octadecadienoic acid ethyl ester	20.681 ± 1.045 ^a^	4.076 ± 0.226 ^b^	80.29	5.303 ± 0.296 ^b^	74.36
Alcohols	71-23-8	1-Propanol	26.82 ± 1.472	26.405 ± 2.904	1.55	25.252 ± 0.733	5.85
	78-83-1	Isobutanol	5.008 ± 2.385	3.707 ± 0.181	25.98	4.999 ± 1.21	0.20
	71-36-3	1-Butanol	19.517 ± 1.195	18.531 ± 1.766	5.05	18.824 ± 0.916	3.55
	137-32-6	2-Methyl-1-butanol	55.222 ± 19.065	ND	100.00	51.846 ± 0.799	6.11
	123-51-3	3-Methyl-1-butanol	67.157 ± 23.711	69.841 ± 26.83	−4.00	62.749 ± 11.473	6.56
	71-41-0	1-Pentanol	1.411 ± 0.071 ^a^	ND	100.00	3.208 ± 0.02 ^b^	−127.32
	111-35-3	3-Ethoxy-1-propanol	0.178 ± 0.118	0.228 ± 0.006	-28.4	0.193 ± 0.004	−8.70
	513-85-9	2,3-Butanediol	56.604 ± 1.782	51.875 ± 2.812	8.35	58.957 ± 9.082	−4.16
	57-55-6	Propylene Glycol	42.403 ± 3.299	39.151 ± 1.773	7.67	43.727 ± 9.455	−3.12
	98-00-0	Furfuryl alcohol	0.353 ± 0.018 ^a^	0.41 ± 0.069 ^a^	−16.12	0.24 ± 0.003 ^b^	32.03
	100-51-6	Benzyl alcohol	0.921 ± 0.052 ^a^	0.895 ± 0.127 ^a^	2.87	1.348 ± 0.013 ^b^	−46.30
	60-12-8	2-Phenylethanol	16.676 ± 0.6 ^b^	14.071 ± 0.664 ^a^	15.62	15.044 ± 0.585 ^a^	9.78
Acids	64-19-7	Acetic acid	271.231 ± 13.652	243.163 ± 11.552	10.35	271.612 ± 53.482	−0.14
	79-09-4	Propanoic acid	15.443 ± 0.617	13.457 ± 0.767	12.86	14.553 ± 2.443	5.76
	107-92-6	Butanoic acid	15.823 ± 1.624	12.824 ± 2.233	18.95	16.1 ± 1.433	−1.75
	142-62-1	Hexanoic acid	2.984 ± 0.455	2.04 ± 1.331	31.64	2.95 ± 0.477	1.13
	79-31-2	2-Methylpropionic acid	3.183 ± 0.134	2.974 ± 0.301	6.58	3.316 ± 0.345	−4.17
	462-95-3	Diethoxymethane	5.986 ± 0.68 ^a^	6.625 ± 1.229 ^a^	−10.68	3.305 ± 0.011 ^b^	44.78
	3842-03-3	1,1-Diethoxy-3-methyl-Butane	31.732 ± 6.51	37.767 ± 1.537	−19.02	34.671 ± 3.508	−9.26
	7789-92-6	1,1,3-Triethoxypropane	0.532 ± 0.089	0.573 ± 0.003	−7.61	0.536 ± 0.026	−0.58
Others	13925-03-6	2-Methyl-6-ethylpyrazine	0.346 ± 0.069	0.326 ± 0.026	5.79	0.411 ± 0.095	−18.78
	14667-55-1	Trimethyl-pyrazine	0.277 ± 0.017	0.514 ± 0.274	−85.35	ND	100.00
	1124-11-4	Tetramethylpyrazine	0.862 ± 0.093 ^a^	1.078 ± 0.16 ^a^	−25.04	1.546 ± 0.05 ^b^	−79.32
	5774-26-5	1,1-Diethoxyacetone	0.783 ± 0.157	0.649 ± 0.09	17.16	0.648 ± 0.008	17.29
	513-86-0	3-Hydroxy-2-butanone	49.245 ± 2.49	44.413 ± 1.79	9.81	46.657 ± 9.59	5.26
	98-01-1	Furfural	98.339 ± 2.181 ^a^	97.37 ± 6.33 ^a^	0.98	112.672 ± 0.981 ^b^	−14.58
	100-52-7	Benzaldehyde	4.565 ± 0.641	3.687 ± 0.475	19.24	3.929 ± 0.674	13.94
	6270-56-0	Furfuryl ethyl ether	2.683 ± 1.077	2.695 ± 1.994	−0.44	2.739 ± 1.583	−2.09
	1192-62-7	2-Acetylfuran	0.647 ± 0.102	0.55 ± 0.031	14.95	0.603 ± 0.055	6.75
	6314-97-2	(2,2-Diethoxyethyl)-Benzene	0.399 ± 0.016 ^a^	0.326 ± 0.013 ^b^	18.21	0.381 ± 0.067 ^ab^	4.53

^1^ The loss percentage is the concentration loss after adsorption as a percentage of the original concentration; ^2^ The loss percentage is the concentration loss of the control group as a percentage of the original concentration; ^3^ ND means not detected. The concentrations of aroma-active compounds in three Baijiu samples, values (means ± SD, *n* = 3) with different letters (^a^,^b^) indicate significantly difference at *p* ≤ 0.05 (Duncan test).

**Table 2:** Changes in volatile compounds before and after Baijiu II adsorption.

	CAS	Baijiu II Volatile Compounds	Original Concentration (mg/L)	After DBP Adsorption (mg/L)	Loss Percentage ^1^ (%)	After Multicomponent Adsorption (mg/L)	Loss Percentage ^2^ (%)	Concentration of Control Group (mg/L)	Loss Percentage ^3^ (%)
Esters	109-94-4	Ethyl formate	0.268 ± 0.001 ^a^	0.298 ± 0.018 ^ab^	−11.28	0.353 ± 0.055 ^b^	−31.82	0.327 ± 0.028 ^ab^	−22.11
	141-78-6	Ethyl acetate	230.616 ± 21.31 ^a^	247.773 ± 5.549 ^ab^	−7.44	282.786 ± 25.112 ^b^	−22.62	264.978 ± 14.433 ^ab^	−14.90
	123-66-0	Ethyl hexanoate	5.746 ± 0.382 ^a^	6.511 ± 0.508 ^b^	−13.32	7.295 ± 0.089 ^c^	−26.96	7.309 ± 0.092 ^c^	−27.21
	106-30-9	Ethyl heptanoate	0.135 ± 0.001	ND ^4^	100.00	0.07 ± 0.012	48.31	0.126 ± 0.052	6.96
	687-47-8	Ethyl *L*-lactate	323.02 ± 19.754	321.378 ± 6.917	0.51	346.249 ± 41.107	−7.19	312.662 ± 23.854	3.21
	106-32-1	Ethyl octanoate	0.265 ± 0.039	0.152 ± 0.075	42.68	0.192 ± 0.089	27.60	0.275 ± 0.011	−3.70
	52089-54-0	Ethyl 2-hydrobutanoate	1.679 ± 0.026	1.823 ± 0.224	−8.58	1.742 ± 0.275	−3.75	2.069 ± 0.355	−23.23
	2441-06-7	Ethyl 2-hydroxy-3-methylbutanoate	4.779 ± 0.128 ^a^	7.056 ± 1.7 ^ab^	−47.65	8.853 ± 2.908 ^b^	−85.25	5.381 ± 1.154 ^a^	−12.60
	10348-47-7	Ethyl 2-hydroxy-4-methyl pentanoate	0.399 ± 0.021	0.422 ± 0.085	−5.89	0.34 ± 0.294	14.69	0.472 ± 0.037	−18.43
	110-38-3	Ethyl decanoate	0.148 ± 0.001	ND	100.00	0.143 ± 0.001	3.57	0.135 ± 0.019	8.97
	101-97-3	Ethyl 2-phenylacetate	0.217 ± 0.001 ^a^	ND	100.00	0.201 ± 0.038 ^a^	7.25	0.251 ± 0.004 ^b^	−15.83
	628-97-7	Ethyl hexadecanoate	2.198 ± 0.749	ND	100.00	ND	100.00	1.86 ± 0.01	15.39
	123-79-5	Hexanedioic acid, dioctyl Ester	5.87 ± 0.011	ND	100.0	ND	100.00	ND	100.00
Alcohols	78-92-2	2-Butanol	23.102 ± 1.199 ^a^	23.001 ± 0.223 a	0.44	25.299 ± 0.891 ^b^	−9.51	22.113 ± 1.14 ^a^	4.28
	71-23-8	1-Propanol	8.469 ± 0.11 ^a^	8.706 ± 0.672 ^a^	−2.80	10.131 ± 1.082 ^b^	−19.63	9.196 ± 0.639 ^a^	−8.59
	78-83-1	Isobutanol	0.12 ± 0.003	ND	100.00	ND	100.00	ND	100.00
	71-36-3	1-Butanol	0.711 ± 0.021	0.72 ± 0.096	-1.29	0.802 ± 0.023	−12.82	0.791 ± 0.1	−11.28
	1565-80-6	(S)-2-methyl-1-Butanol	1.448 ± 0.204	2.161 ± 0.091	-49.24	1.951 ± 0.741	−34.74	2.185 ± 0.11	−50.90
	123-51-3	3-Methyl-1-butanol	2.327 ± 0.084 ^a^	2.706 ± 0.311 ^ab^	-16.28	3.356 ± 0.898 ^b^	−44.22	3.244 ± 0.299 ^ab^	−39.40
	2517-43-3	3-Methoxy-1-butanol	0.118 ± 0.002	ND	100.00	ND	100.00	ND	100.00
	513-85-9	2,3-Butanediol	15.039 ± 2.898	14.463 ± 1.625	3.83	17.606 ± 1.613	−17.07	15.753 ± 0.676	−4.75
	57-55-6	Propylene glycol	2.816 ± 0.321 ^a^	4.578 ± 0.023 ^b^	-62.59	4.968 ± 0.777 ^b^	−76.44	1.95 ± 0.358 ^a^	30.75
	60-12-8	2-Phenylethanol	3.106 ± 0.446	3.034 ± 0.425	2.32	3.364 ± 0.317	−8.31	3.491 ± 0.405	−12.40
	98-00-0	Furfuryl alcohol	1.667 ± 0.14	1.736 ± 0.289	-4.16	1.88 ± 0.408	−12.80	ND	100.00
Else	64-19-7	Acetic acid	238.038 ± 20.511	232.966 ± 5.879	2.13	248.063 ± 5.927	−4.21	231.039 ± 15.955	2.94
	79-09-4	Propanoic acid	0.847 ± 0.374	0.618 ± 0.269	27.07	0.677 ± 0.216	20.11	0.557 ± 0.14	34.27
	107-92-6	Butanoic acid	4.136 ± 0.469 ^a^	3.783 ± 0.014 ^ab^	8.54	3.296 ± 0.321 ^b^	20.31	3.036 ± 0.448 ^b^	26.60
	142-62-1	Hexanoic acid	7.616 ± 1.659 ^a^	0.751 ± 0.029 ^b^	90.14	7.43 ± 0.536 ^a^	2.45	7.893 ± 0.305 ^a^	−3.63
	98-01-1	Furfural	5.407 ± 0.395	4.645 ± 1.65	14.09	4.48 ± 0.581	17.14	5.885 ± 0.648	−8.85
	100-52-7	Benzaldehyde	0.632 ± 0.11	0.668 ± 0.031	−5.73	0.778 ± 0.196	−23.14	0.847 ± 0.11	−34.06
	513-86-0	3-Hydroxy-2-butanone	2.617 ± 0.459	2.77 ± 0.19	−5.85	2.896 ± 0.077	−10.67	2.98 ± 0.125	−13.88
	14667-55-1	Trimethyl-pyrazine	0.458 ± 0.001	ND	100.00	ND	100.00	ND	100.00
	6314-97-2	(2,2-Diethoxyethyl)-Benzene	0.183 ± 0.016	ND	100.00	ND	100.00	0.204 ± 0.005	−11.46

^1^ The loss percentage is the loss concentration after DBP adsorption as a percentage of the original concentration; ^2^ The loss percentage is the loss concentration after multicomponent adsorption as a percentage of the original concentration; ^3^ The loss percentage is the loss concentration of control group as a percentage of the original concentration; ^4^ ND means not detected. The concentrations of aroma-active compounds in four Baijiu samples, values (means ± SD, *n* = 3) with different letters (^a^, ^b^ and ^c^) indicate significantly difference at *p* ≤ 0.05 (Duncan test).

## Data Availability

Data is contained within the article or supplementary material.

## Supplementary Materials

The following supporting information can be downloaded at: https://www.mdpi.com/article/10.3390/foods11142114/s1, Figure S1: GC-MS ion chromatography of multicomponent PAEs; Figure S2: SEM images of the AC; Figure S3: FTIR of AC before and after adsorption of DBP; Figure S4: N_2_ adsorption-desorption results of AC. (a) N_2_ adsorption-desorption isotherms; (b) Pore size distribution; Figure S5Adsorption model; Figure S6: Forms of DBP in acid-base environments. (a) Acetic acid environment; (b) Pyridine environment; Figure S7: Isoelectric point of AC; Figure S8: Intra-particle diffusion model; Figure S9: The radial distribution function (RDF) plots between different particles. (a) RDF of DBP and AC; (b) RDF of DBP and solution; Figure S10: Concentration distribution of DBP in: (a) water, (b) 50 v% ethanol, (c) 100 v% ethanol; Figure S11: Introduction to the whole idea and method of this paper; Table S1: The GC-MS peak time schedule of multicomponent PAEs; Table S2: AC surface and pore structure data; Table S3: Kinetic fitting parameters of DBP adsorption on AC; Table S4: Intra-particle diffusion equation parameters; Table S5: Adsorption isotherm model parameters of DBP; Table S6: AC adsorption of DBP thermodynamic model parameters; Table S7: Physico-chemical properties of five PAEs concerned in this paper; Table S8: Molecular size table of five PAEs concerned in this paper; Table S9: Interaction energies of different simulated composite systems; Table S10: The position of the highest peak on both sides; Table S11: Adsorption capacity comparison of PAEs; Table S12: Adsorption capacity of AC for DBP in the manuscript; Table S13: Adsorption capacity of AC for multicomponent PAEs in the manuscript; Table S14: Adsorption capacity comparison of DBP in Baijiu. References [8,28,42,43,44,45,46,47,48,49,50,51,52,53,54,55,56] are cited in the supplementary materials.

## References

[1] Ambe K., Sakakibara Y., Sakabe A., Makino H., Ochibe T., Tohkin M. (2019). Comparison of the developmental/reproductive toxicity and hepatotoxicity of phthalate esters in rats using an open toxicity data source. J. Toxicol. Sci..

[2] Hou H., Min Y., Liu X., Wang P., Zhou Z., Liu D. (2021). Occurrence and migration of phthalates in adhesive materials to fruits and vegetables. J. Hazard. Mater..

[3] U.S.E.P. Agency, U.S. Environmental Protection Agency (2012). Phthalates Action Plan (Revised).

[4] Gao M., Dong Y., Zhang Z., Song W., Qi Y. (2017). Growth and antioxidant defense responses of wheat seedlings to di-n-butyl phthalate and di (2-ethylhexyl) phthalate stress. Chemosphere.

[5] Xu Y., Zhao J., Huang H., Guo X., Li X., Zou W., Li W., Zhang C., Huang M. (2022). Biodegradation of phthalate esters by Pantoea dispersa BJQ0007 isolated from Baijiu. J. Food Compos. Anal..

[6] Moreno J.D., Poznyak T., Chairez I., Dorantes-Rosales H.J. (2020). Effect of the type of soil on dimethyl phthalate degradation by ozone. J. Environ. Manag..

[7] Deng D., Wu X., Li M., Qian S., Tang B., Wei S., Zhang J. (2020). Electrochemical degradation of three phthalate esters in synthetic wastewater by using a Ce-doped Ti/PbO_2_ electrode. Chemosphere.

[8] Ye H., Zhao B., Zhou Y., Du J., Huang M. (2021). Recent advances in adsorbents for the removal of phthalate esters from water: Material, modification, and application. Chem. Eng. J..

[9] Sullivan K.F., Atlas E.L., Giam C.S. (1982). Adsorption of phthalic acid esters from seawater. Environ. Sci. Technol..

[10] Medellin-Castillo N.A., Ocampo-Pérez R., Leyva-Ramos R., Sanchez-Polo M., Rivera-Utrilla J., Méndez-Díaz J.D. (2013). Removal of diethyl phthalate from water solution by adsorption, photo-oxidation, ozonation and advanced oxidation process (UV/H_2_O_2_, O_3_/H_2_O_2_ and O_3_/activated carbon). Sci. Total Environ..

[11] Bouhamidi Y., Kaouah F., Nouri L., Boumaza S., Trari M., Bendjama Z. (2018). Kinetic, thermodynamic, and isosteric heat of dibutyl and diethyl phthalate removal onto activated carbon from *Albizzia julibrissin pods*. Part. Sci. Technol..

[12] Jing F., Pan M., Chen J. (2018). Kinetic and isothermal adsorption-desorption of PAEs on biochars: Effect of biomass feedstock, pyrolysis temperature, and mechanism implication of desorption hysteresis. Environ. Sci. Pollut. Res..

[13] Qureshi U.A., Solangi A.R., Memon S.Q., Taqvi S.I.H. (2014). Utilization of Pine Nut Shell derived carbon as an efficient alternate for the sequestration of phthalates from aqueous system. Arab. J. Chem..

[14] Gao L., Tang Y., Wang C., Yao L., Zhang J., Gao R., Tang X., Chong T., Zhang H. (2019). Highly-efficient amphiphilic magnetic nanocomposites based on a simple sol-gel modification for adsorption of phthalate esters. J. Colloid Interface Sci..

[15] Diao X., Hu Y., He Y., Quan F., Wei C. (2017). Preparation of 3,3,3-trifluoropropyl functionalized hydrophobic mesoporous silica and its outstanding adsorption properties for dibutyl phthalate. RSC Adv..

[16] Khan N.A., Jung B.K., Hasan Z., Jhung S.H. (2015). Adsorption and removal of phthalic acid and diethyl phthalate from water with zeolitic imidazolate and metal–organic frameworks. J. Hazard. Mater..

[17] Yang R., Liu Y., Yan X., Liu S., Zheng H. (2016). An Effective Method for the Synthesis of Yolk–Shell Magnetic Mesoporous Carbon–Surface Molecularly. J. Mater. Chem. A.

[18] Mok C.F., Ching Y.C., Osman N.A.A., Muhamad F., Hai N.D., Choo J.H., Hassan C.R. (2020). Adsorbents for removal of cationic dye: Nanocellulose reinforced biopolymer composites. J. Polym. Res..

[19] Jin Z., Xiao S., Dong H., Xiao J., Tian R., Chen J., Li Y., Li L. (2022). Adsorption and catalytic degradation of organic contaminants by biochar: Overlooked role of biochar’s particle size. J. Hazard. Mater..

[20] Sun H., Jin Z., Yang C., Akkermans R.L.C., Robertson S.H., Spenley N.A., Miller S., Todd S.M. (2016). COMPASS II: Extended coverage for polymer and drug-like molecule databases. J. Mol. Model..

[21] Wang Z., Wang Y., Zhu T., Wang J., Huang M., Wei J., Ye H., Wu J., Zhang J., Meng N. (2022). Characterization of the key odorants and their content variation in Niulanshan Baijiu with different storage years using flavor sensory omics analysis. Food Chem..

[22] Méndez-Díaz J.D., Daiem M.M.A., Rivera-Utrilla J., Sánchez-Polo M., Bautista-Toledo I. (2012). Adsorption/bioadsorption of phthalic acid, an organic micropollutant present in landfill leachates, on activated carbons. J. Colloid Interface Sci..

[23] Shaida M.A., Dutta R.K., Sen A.K. (2018). Removal of diethyl phthalate via adsorption on mineral rich waste coal modified with chitosan. J. Mol. Liq..

[24] Wang X., Zhao K., Yang B., Chen T., Li D., Wu H., Wei J., Wu X. (2016). Adsorption of dibutyl phthalate in aqueous solution by mesoporous calcium silicate grafted non-woven polypropylene. Chem. Eng. J..

[25] Zhang H., Fang D., Kong Z., Wei J., Wu X., Shen S., Cui W., Zhu Y. (2018). Enhanced adsorption of phthalic acid esters (PAEs) from aqueous solution by alkylbenzene-functionalized polypropylene nonwoven and its adsorption mechanism insight. Chem. Eng. J..

[26] Hu Y., Gu Z., Li W., Xu C.C. (2020). Alkali-catalyzed liquefaction of pinewood sawdust in ethanol/water co-solvents. Biomass Bioenergy.

[27] Wood A.L., Bouchard D.C., Brusseau M.L., Rao P.S.C. (1990). Cosolvent effects on sorption and mobility of organic contaminants in soils. Chemosphere.

[28] Wypych A. (2017). Databook of Plasticizers.

[29] Gao M., Zhang Y., Gong X., Song Z., Guo Z. (2018). Removal mechanism of di-n-butyl phthalate and oxytetracycline from aqueous solutions by nano-manganese dioxide modified biochar. Environ. Sci. Pollut. Res..

[30] Mohan S.V., Shailaja S., Krishna M.R., Sarma P.N. (2007). Adsorptive removal of phthalate ester (Di-ethyl phthalate) from aqueous phase by activated carbon: A kinetic study. J. Hazard. Mater..

[31] Cavalcante A.d., Chelli R. (2021). Polarizability relaxation in water/ethanol mixtures. J. Mol. Liq..

[32] Zhang Y., Chen G., Gu J., Ma C., Li L., Zhu C., Gao H., Wang C., Shang Y., Yang Z. (2020). A theoretical study on dimerization and dissociation of acetic acid in ethanol solvent. Comput. Theor. Chem..

[33] Zhang C., Yang X. (2005). Molecular dynamics simulation of ethanol/water mixtures for structure and diffusion properties. Fluid Phase Equilibr..

[34] Wang S., Cann N.M. (2007). Polarizable and flexible model for ethanol. J. Chem. Phys..

[35] Liu Y., Luo X., Shen Z., Lu J., Ni X. (2006). Studies on Molecular Structure of Ethanol-Water Clusters by Fluorescence Spectroscopy. Opt. Rev..

[36] Guo L., Xu Y., Zhuo M., Liu L., Xu Q., Wang L., Shi C., Ye B., Fan X., Chen W. (2018). Highly efficient removal of Gd (III) using hybrid hydrosols of carbon nanotubes/graphene oxide in dialysis bags and synergistic enhancement effect. Chem. Eng. J..

[37] Ma S., Jing F., Sohi S.P., Chen J. (2019). New insights into contrasting mechanisms for PAE adsorption on millimeter, micron- and nano-scale biochar. Environ. Sci. Pollut. Res. Int..

[38] Abdul G., Zhu X., Chen B. (2017). Structural characteristics of biochar-graphene nanosheet composites and their adsorption performance for phthalic acid esters. Chem. Eng. J..

[39] Yang H., Ye S., Zeng Z., Zeng G., Tan X., Xiao R., Wang J., Song B., Du L., Qin M. (2020). Utilization of biochar for resource recovery from water: A review. Chem. Eng. J..

[40] Jalali A.M., Shariatinia Z., Taromi F.A. (2017). Desulfurization efficiency of polydimethylsiloxane/silica nanoparticle nanocomposite membranes: MD simulations. Comp. Mater. Sci..

[41] Jiang X., Wang W., Yu G., Deng S. (2021). Contribution of Nanobubbles for PFAS Adsorption on Graphene and OH- and NH_2_-Functionalized Graphene: Comparing Simulations with Experimental Results. Environ. Sci. Technol..

[42] Wang J., Zhang B., Wu Q., Jiang X., Liu H., Wang C., Huang M., Wu J., Zhang J., Yu Y. (2022). Sensomics-assisted flavor decoding of coarse cereal Huangjiu. Food Chem..

[43] Wang J., Yuan C., Gao X., Kang Y., Huang M., Wu J., Liu Y., Zhang J., Li H., Zhang Y. (2020). Characterization of key aroma compounds in Huangjiu from northern China by sensory-directed flavor analysis. Food Res. Int..

[44] TKhan A., Mukhlif A.A., Khan E.A. (2019). Uptake of Cu^2+^ and Zn^2+^ from simulated wastewater using muskmelon peel biochar: Isotherm and kinetic studies. Egypt. J. Basic Appl. Sci..

[45] Ho Y.S., McKay G. (1999). Pseudo-second order model for sorption processes. Process. Biochem..

[46] Huang H., Fan Y., Wang J., Gao H., Tao S. (2013). Adsorption kinetics and thermodynamics of water-insoluble crosslinked β-cyclodextrin polymer for phenol in aqueous solution. Macromol. Res..

[47] Khan A.A., Singh R.P. (1987). Adsorption thermodynamics of carbofuran on Sn (IV) arsenosilicate in H^+^, Na^+^ and Ca^2+^ forms. Colloids Surf..

[48] Wu Y., Si Y., Zhou D., Gao J. (2015). Adsorption of diethyl phthalate ester to clay minerals. Chemosphere.

[49] Lu L., Wang J., Chen B. (2018). Adsorption and desorption of phthalic acid esters on graphene oxide and reduced graphene oxide as affected by humic acid. Environ. Pollut..

[50] Brunauer S., Deming L.S., Deming W.E., Teller E. (1940). On a Theory of the van der Waals Adsorption of Gases. J. Am. Chem. Soc..

[51] Song T., Liao J., Xiao J., Shen L. (2015). Effect of micropore and mesopore structure on CO_2_ adsorption by activated carbons from biomass. New Carbon Mater..

[52] Groen J.C., Peffer L.A.A., Pérez-Ramírez J. (2003). Pore size determination in modified micro- and mesoporous materials. Pitfalls and limitations in gas adsorption data analysis. Microporous Mesoporous Mater..

[53] Kiso Y., Kon T., Kitao T., Nishimura K. (2001). Rejection properties of alkyl phthalates with nanofiltration membranes. J. Membr. Sci..

[54] Tang P., Zhou J., Yu G. (2017). Application of Coconut Shell Activated Carbon in the Treatment of Plasticizer (DBP) in Jiangxiang Baijiu. Liquor-Mak. Sci. Technol..

[55] Wang Y., Yang W., Zheng M. (2014). Research on the Processing Methods of Plasticizer in Baijiu (Liquor). Liquor-Mak. Sci. Technol..

[56] Dong R., Zhang J., Yang S. (2022). Effect of DBP Removal by Activated Carbon on the Quality of Baijiu. Liquor-Mak. Sci. Technol..

