# Supplementary material for: Adsorption of Phthalate Acid Esters by Activated Carbon: The Overlooked Role of the Ethanol Content
Source: Foods. 2022 Jul 15;11(14):2114. doi: 10.3390/foods11142114 (PMC9323295; doi:10.3390/foods11142114)
Supplement: Supplementary file 1 [file foods-11-02114-s001.zip › foods-1806075-supplementary.pdf]

foods
MDPI

Supplementary Materials

Adsorption of Phthalate Acid Esters by Activated Carbon: The Overlooked Role of the Ethanol Content

Yuanhao Zhou [1,2], Bingyu Zhao [1,2], Lingxuan Wang [1,2], Ting Li [1,2], Hong Ye [1,2,*], Shuangyang Li [1,2], Mingquan Huang [1,2] and Xianren Zhang [3]

[1] Key Laboratory of Brewing Molecular Engineering of China Light Industry, Beijing Technology and Business University, Beijing 100048, China; zhouyuanhao97@163.com (Y.Z.); zhaoby98@163.com (B.Z.); yqdszhdapxuan@163.com (L.W.); liting120902@163.com (T.L.); lishuangyang@th.btbu.edu.cn (S.L.); huangmq@th.btbu.edu.cn (M.H.)
[2] Beijing Laboratory of Food Quality and Safety, Beijing Technology and Business University, Beijing 100048, China
[3] State Key Laboratory of Organic-Inorganic Composites, Beijing University of Chemical Technology, Beijing 100029, China; zhangxr@mail.buct.edu.cn
* Correspondence: yehcn@163.com

Text S1. Gas Chromatography-Mass Spectrometry (GC-MS) program and analysis

A Thermo Fisher Trace 1310 gas chromatograph equipped with single quandrupole (ISQ) mass spectrometer (GC–MS) was used in this study. The PAEs were separated using a polar DB-FFAP column (30 m×0.25 mm×0.25 μm). The carrier gas was helium (99.999%) at a constant flow rate of 1.5 mL/min and the inlet temperature was 250 °C. The sample (1.0 μL) was injected in splitless mode. The temperature program was as follows: the initial oven temperature was 60 °C for 3 min, then ramped to 220 °C at 20°C/min and held for 5 min, then finally ramped to 250 °C at 10 °C/min and held for 15 min to detect single component DBP. For multi-component detection, the final temperature rose to 250 °C and held 25 min.

The mass spectrometry conditions were as follows: the temperature of the ion source temperature was 230 °C, and the transmission line temperature was 280 °C. Mass spectra were generated in the EI mode at 70 eV, and quantitative ion scanning mode m/z=149 (DEP, DPP, DBP, DEHP and DnOP) and m/z=163 (DMP) were used. The retention time of different PAEs were shown in Table S1 and Figure S1.

Table S1. The GC-MS peak time schedule of multicomponent PAEs.

PAEs	Time(min)
DMP	12.91
DEP	13.50
DPP	15.03
DBP	16.91
DEHP	24.85
DnOP	35.97

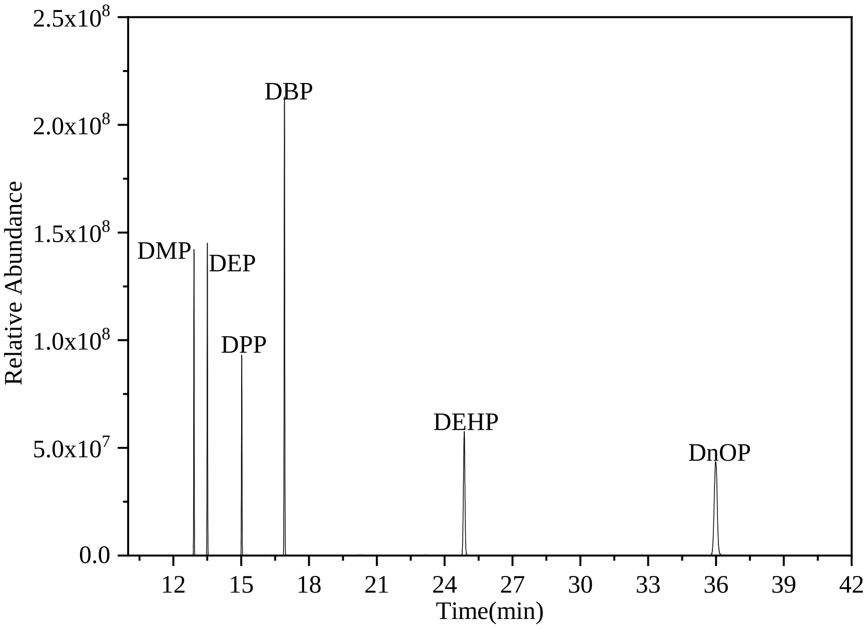

Figure S1. GC-MS ion chromatography of multicomponent PAEs.

Text S2. Identification of volatile compounds by GC–MS and quantitative analysis of volatile compounds

Anhydrous sodium sulfate (0.20 g) was added to 1.0 mL of Baijiu samples, and the samples were spiked with 4-octanol to a final concentration of 82.0 mg/L. After 6 h, the samples passed through nylon membranes (0.45 μm), and 1.0 μL of the filtered samples was injected into GC-MS in spitless. A Thermo Fisher Trace 1310 gas chromatograph equipped with a single quadrupole (ISQ) mass spectrometer (GC–MS) was used in this study. Each sample was analyzed using a DB-FFAP column (60 m × 0.25 mm i.d., 0.25 μm film thickness; J&W). Helium (99.999%) was used as the carrier gas at a flow rate of 1.5 mL/min. The oven was initially held at 40 °C, then increased to 50 °C at 10 °C/min and held for 5.0 min, then increased to 80 °C at 3 °C/min and kept for 5.0 min, and finally ramped to 240 °C at 5 °C/min and held for 5.0 min. The MS conditions were as follows: EI mode, 70 eV; quadrupole temperature, 150 °C; ion source temperature, 240 °C. The identification of aroma compounds was conducted in a full scan mode and the mass range (m/z) was set from 45 to 400.

Qualitative analysis of volatile compounds in Jiang-flavor style Baijiu was conducted using MS and retention index (RI) comparison. The reported RIs and standard mass spectra were from the NIST library 2017. The actual RI value was calculated according to the reported formula[42] using the peak time of the target compound and the peak time of a series of n-alkanes (C7-C30) under the same GC-MS parameters.

For the detection of relative content changes of volatile compounds, a semi-quantify method was used in the study. 4-Octanol (concentration 82 mg/L) was used as the internal standard compound to semi-quantify the volatile compounds in Jiang-flavor style Baijiu. The relative concentrations of all volatile compounds were calculated by Equations (Eqs.) (1):

$$C_X = \frac{A_O}{A_X} \times C_O \qquad \text{(S1)}$$

Where Ao and Ax represent the peak areas of the internal standard compound and the unknown compound, respectively; Co and Cx represent the concentrations of the internal standard compound and the unknown compound (mg/L), respectively[43].

Text S3. Kinetic models, intra-particle diffusion model, adsorption isothermal models and thermodynamic models

The amount of PAEs adsorbed on the adsorbent was calculated using Eqs. (2):

$$Q = \frac{(C_0 - C_t)\ V}{m} \tag{S2}$$

Where Q is the equilibrium adsorption capacity (mg/g); C_0 and C_t are the initial and equilibrium concentrations (mg/L) of PAEs respectively; V is the volume of solution (L); and m is the mass of the used AC (g).

Pseudo-first order, pseudo-second order and intra-particle diffusion kinetic models were used to investigate sorption mechanism using Eqs. (3), (4) and (5), respectively:

$$Q_t = Q_e[1 - e^{(-k_1 t)}] \tag{S3}$$

$$\frac{t}{Q_t} = \frac{1}{k_2 {Q_e}^2} + \frac{t}{Q_e} \tag{S4}$$

$$Q_t = k_{ip} t^{0.5} + C \tag{S5}$$

Where k_1 and k_2 are the rate constants of pseudo-first order and pseudo-second order kinetic models (mg/ (g·h)); k_{ip} is the diffusion rate constant (mg/(g·$h^{0.5}$)); C is the constant related to boundary layer thickness[44,45].

Langmuir and Freundlich models were applied to fit the isotherm adsorption using Eqs. (6) and (7), respectively:

$$\frac{C_e}{Q_e} = \frac{1}{Q_m K_l} + \frac{C_e}{Q_m} \tag{S6}$$

$$ln\, Q_e = ln\, K_f + \frac{1}{n} ln\, C_e \tag{S7}$$

Where C_e (mg·L^{-1}): the equilibrium adsorbate concentration; Q_e (mg·g^{-1}): the equilibrium adsorption capacity; Q_m (mg·g^{-1}): the maximum adsorption capacity; K_l is Langmuir equilibrium coefficients representing adsorption capacity; K_f and n are Freundlich equilibrium coefficients representing adsorption capacity and linearity[46].

Van't Hoff equation was applied to fit the thermodynamic adsorption using Eqs. (8) and (9), respectively:

$$\Delta G = -RT \ln K \tag{S8}$$

$$\ln K = \frac{\Delta S}{R} - \frac{\Delta H}{RT} \tag{S9}$$

Where R is the gas constant (8.314 J/mol K); T is the thermodynamic temperature (K); K is the thermodynamic equilibrium constant. ΔH and ΔS can be calculated according to the slope of the linear equation obtained by fitting lnK to $\frac{1}{RT}$ at different temperatures[47].

Text S4. Characterization of AC

The SEM image of AC was shown in Fig. S2 and the particle size of AC is approximately 400 μm. The AC has relatively uniform particle size and irregular shape. Its surface is relatively rough, the structure is loose, and there is a clear layered structure.

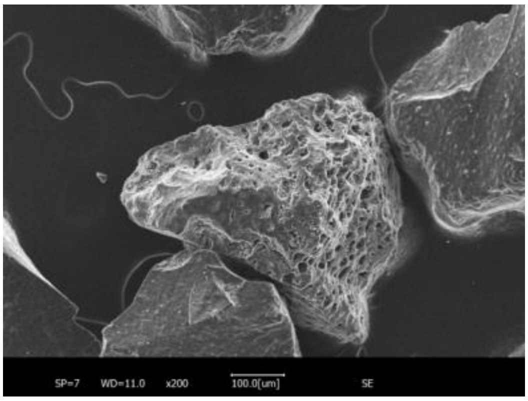

Figure S2. SEM images of the AC.

Fig. S3 shows the FTIR spectra of the original AC and AC after adsorption at ethanol contents of 50 and 100 v% with DBP of 10 mg/L. The peaks at 1113, 1630, and 3440 cm^{-1} were assigned to C-O-C stretching, C=C, and O-H asymmetric stretching, respectively. The -OH stretching vibration peak at 3440 cm^{-1} shifted to 3437 cm^{-1} after adsorption, indicating that -OH participated in the adsorption reaction and formed a hydrogen bond with DBP[48]. The C=C peak at 1630 cm^{-1} shifted to 1623 cm^{-1} and 1624 cm^{-1} after adsorption, indicating that the conjugated structure on the AC surface had π–π electron–donor–acceptor (EDA) interaction with the benzene ring of DBP[49]. The C-O-C peak at 1113 cm^{-1} shifted to 1110 cm^{-1} and 1104 cm^{-1} after adsorption, which showed that the oxygenous groups on the AC surface were also involved in DBP asorption and changed the strength of the functional groups.

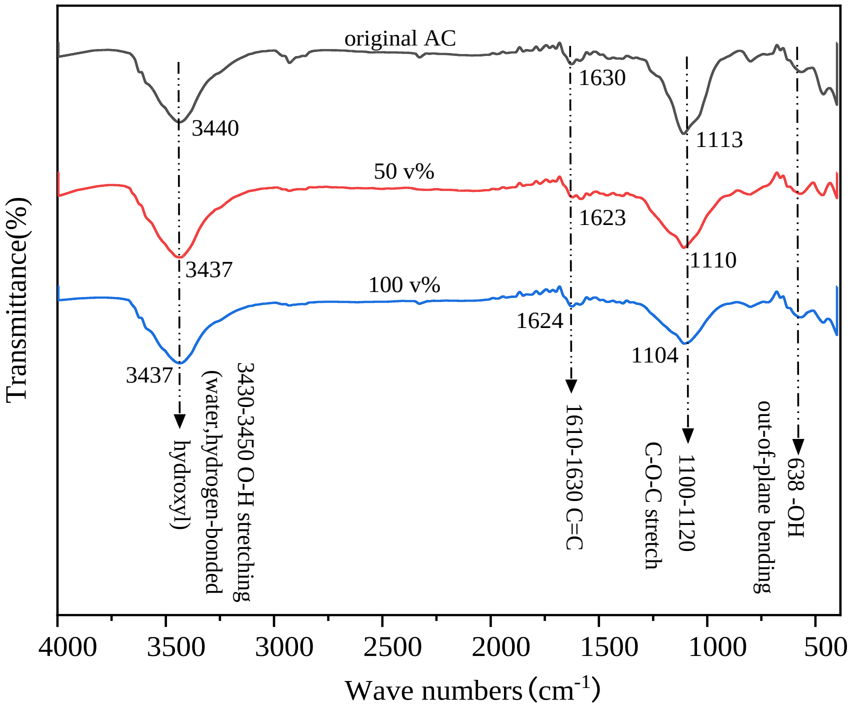

Figure S3. FTIR of AC before and after adsorption of DBP.

The pore structure is very important for adsorption, and the N_2 adsorption-desorption isotherms and the pore size distribution (PSD) of AC are shown in Fig. S4. The adsorption-desorption curve of AC conforms to the Langmuir adsorption isotherm of class I[50].When the relative pressure (P/P_0) was low, the adsorption capacity increased sharply, indicating that the AC had a well-developed microporous structure. As P/P_0 increased, the micropore filling reached saturation and the adsorption isotherm tended to equilibrate[51]. The adsorption-desorption curve exhibited hysteresis, and the desorption curve of the hysteresis loop interval was always higher than the adsorption curve. This was because capillary condensation occurred during adsorption, and capillary evaporation during desorption separated the vapor from the capillary condensation phase[52]. Therefore, the desorption curve can be used to obtain a more accurate PSD. AC has peaks in the micropore and mesopore regions, as shown in Fig. S4(b).

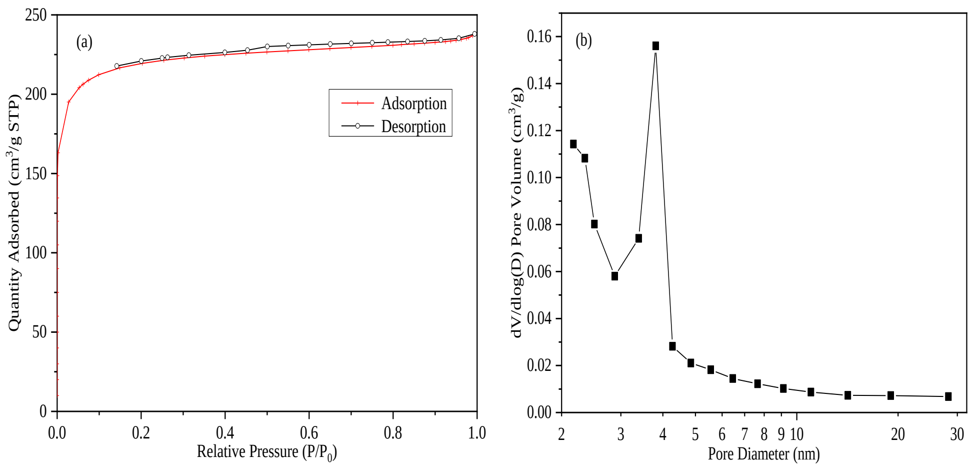

Figure S4. N_2 adsorption-desorption results of AC. (a) N_2 adsorption-desorption isotherms; (b) Pore size distribution.

Table S2. AC surface and pore structure data.

S_{BET} (cm²/g)	S_{micro} (cm²/g)	V_{total} (cm³/g)	V_{micro} (cm³/g)	D_A (nm)
656.94	615.84	0.37	0.32	3.50

As shown in Table S2, the specific surface area of micropores (S_{micro}) accounted for 93.74% of the total specific surface area (S_{BET}), and the micropore volume (v_{micro}) accounted for 86.49% of the total pore volume (v_{total}). According to the regulations of the International Union of Pure and Applied Chemistry, pores smaller than 2 nm are micropores, pores between 2 and 50 nm are mesopores, and pores larger than 50 nm are macropores. The average pore diameter of the AC is mesoporous. The large specific surface area and abundant pore structure endow AC with good adsorption performance.

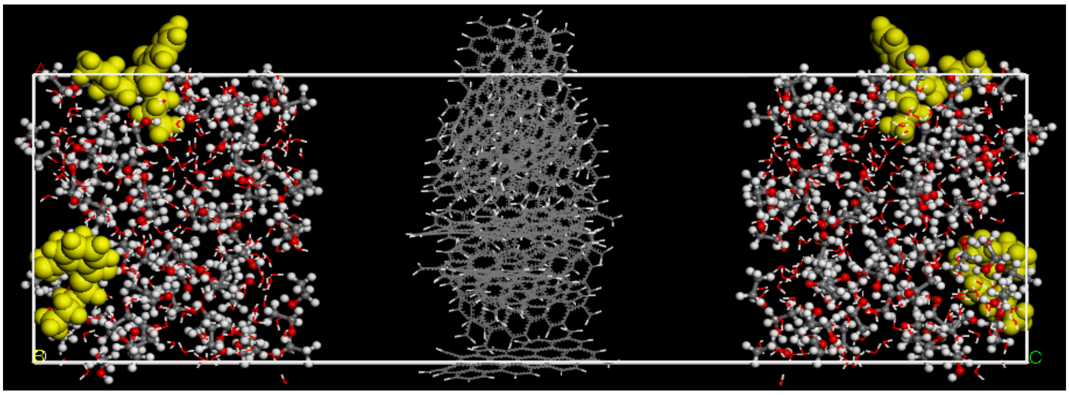

Figure S5. Adsorption model.

AC and water are in line style; ethanol is in ball-and-stick style; DBP is displayed in yellow and CPK style.

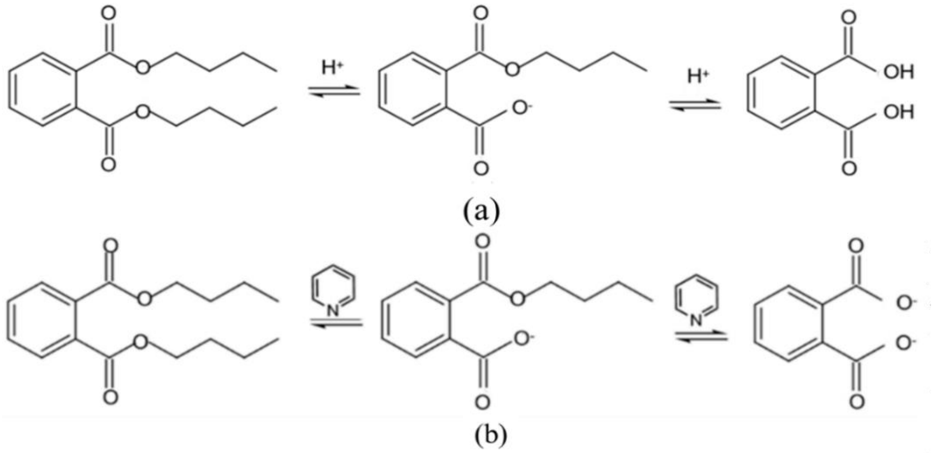

Figure S6. Forms of DBP in acid-base environments. (a) Acetic acid environment; (b) Pyridine environment.

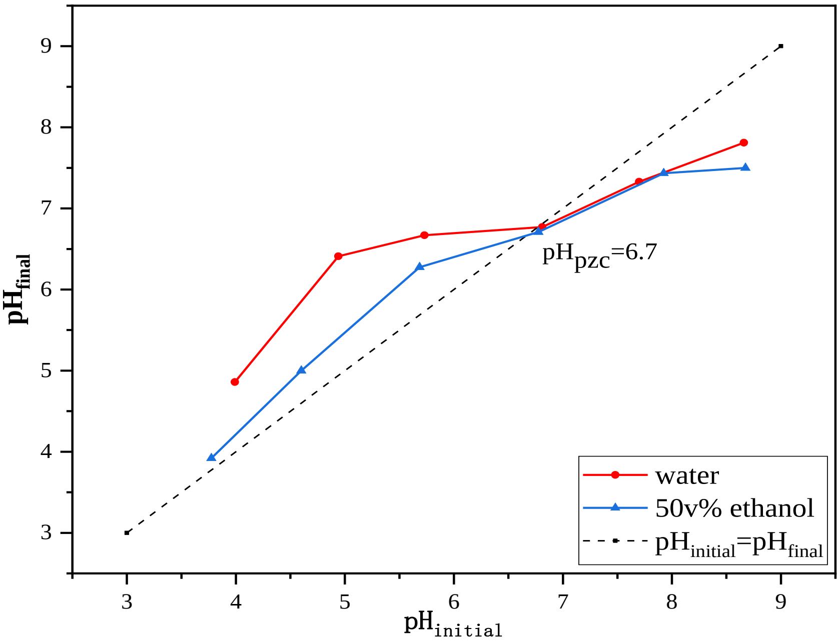

Figure S7. Isoelectric point of AC.

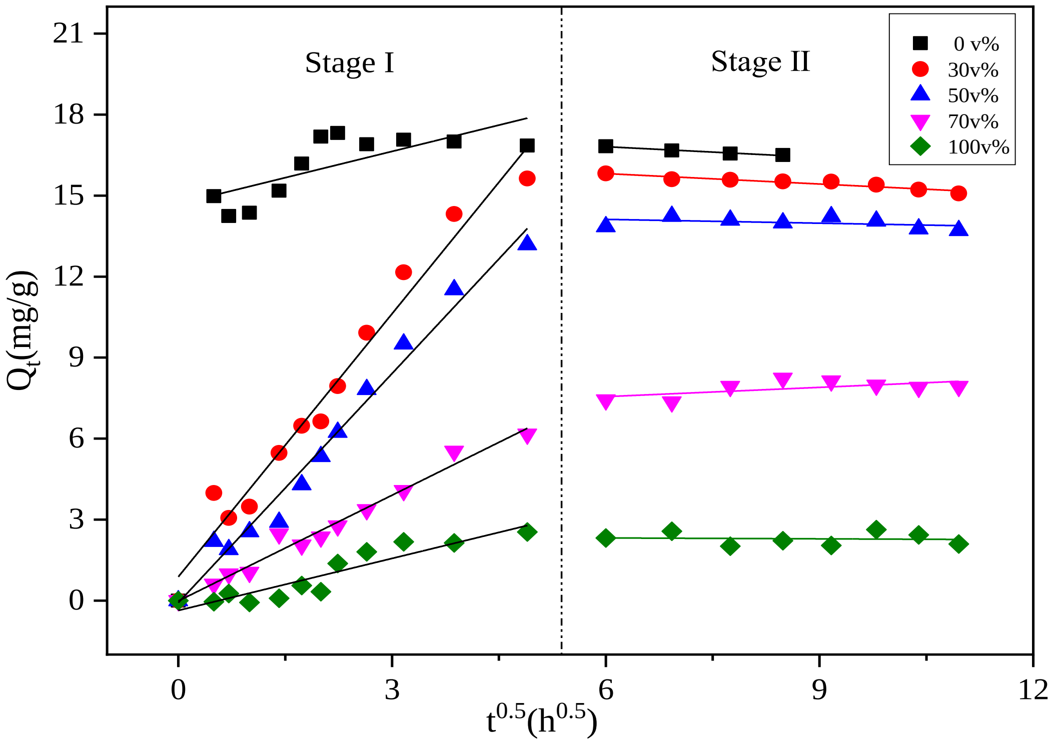

Figure S8. Intra-particle diffusion model.

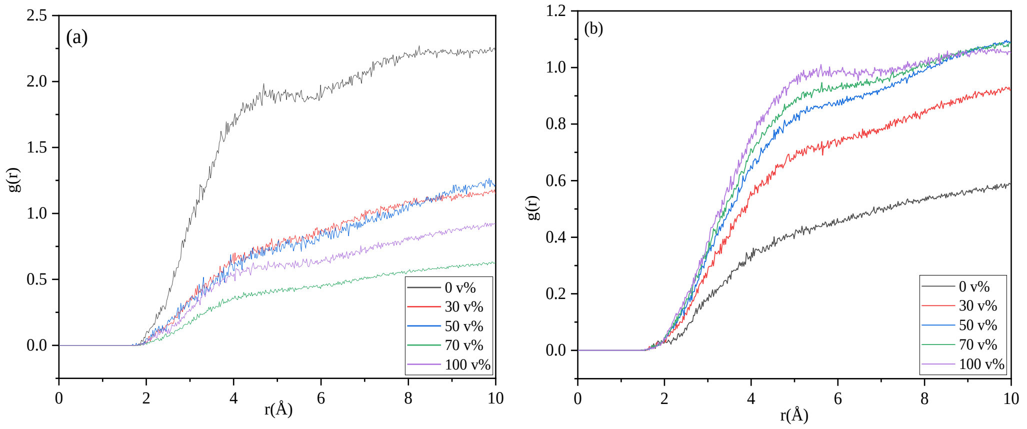

Figure S9. The radial distribution function (RDF) plots between different particles. (a) RDF of DBP and AC; (b) RDF of DBP and solution.

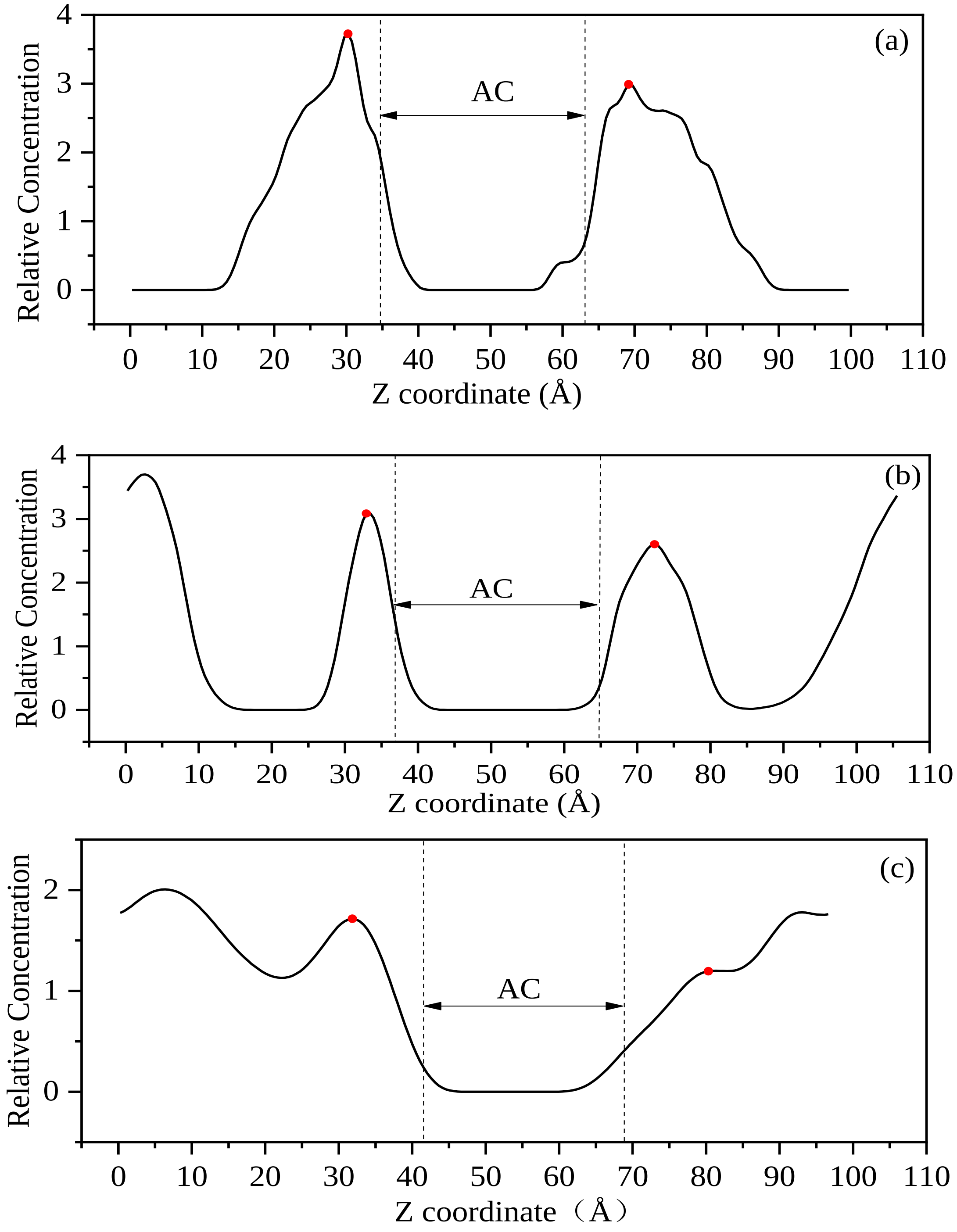

Figure S10. Concentration distribution of DBP in: (a) water, (b) 50 v% ethanol, (c) 100 v% ethanol.

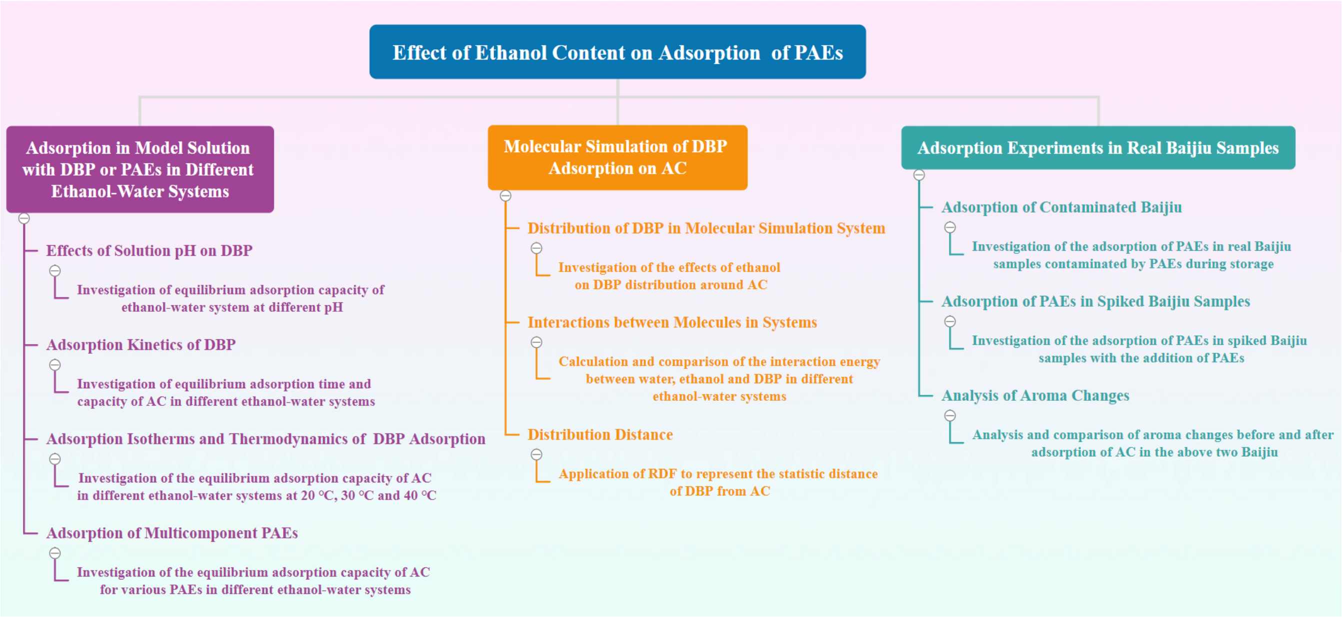

Figure S11. Introduction to the whole idea and method of this paper.

Table S3. Kinetic fitting parameters of DBP adsorption on AC.

Ethanol volume fraction (v%)	$Q_{e,exp}$ (mg/g)	Pseudo-first order kinetic parameters			Pseudo-second order kinetic parameters		
		$Q_{e,calc}$ (mg/g)	K_1 (h^{-1})	R^2(adj)	$Q_{e,calc}$ (mg/g)	K_2 (mg/(g·h))	R^2(adj)
0	16.82	16.39	8.69	0.9484	16.69	1.18	0.9702
30	15.49	15.47	0.16	0.9627	15.99	0.02	0.9440
50	13.99	13.99	0.12	0.9886	15.38	0.01	0.9813
70	8.02	7.89	0.08	0.9789	8.94	0.01	0.9848
100	2.24	2.34	0.17	0.9468	2.53	0.09	0.9131

Table S4. Intra-particle diffusion equation parameters.

Ethanol volume fraction (v%)	Stage I			Stage II		
	k_{ip} (mg/(g·$h^{0.5}$))	C	R^2(adj)	k_{ip} (mg/(g·$h^{0.5}$))	C	R^2(adj)
0	2.26	12.32	0.9477	-0.09	17.28	0.9582
30	3.25	0.88	0.9680	-0.13	16.59	0.8845
50	2.83	-0.079	0.9795	-0.05	14.40	0.0285
70	1.31	-0.015	0.9761	0.11	6.87	0.3020
100	0.70	-0.44	0.8102	-0.01	2.39	-0.1592

Table S5. Adsorption isotherm model parameters of DBP.

Ethanol volume fraction (v%)	temperature (°C)	Langmuir			Freundlich		
		Q_m (mg/g)	K_L	R^2(adj)	K_F	n	R^2(adj)
	20	161.29	0.38	0.9786	42.20	1.69	0.9447
30	30	163.93	0.36	0.9689	43.12	1.75	0.9098
	40	149.25	0.65	0.9888	60.91	2.41	0.9612
	20	78.13	0.13	0.9956	15.90	2.40	0.9667
50	30	81.97	0.13	0.9993	16.32	2.38	0.9886
	40	64.10	0.26	0.9963	20.98	3.22	0.9445
	20	36.23	0.06	0.9744	5.75	2.46	0.9734
70	30	47.62	0.02	0.9853	2.37	1.60	0.9827
	40	51.02	0.02	0.9972	2.19	1.51	0.9971
	20	21.23	0.03	0.9915	2.34	2.24	0.9915
100	30	19.65	0.04	0.9991	2.26	2.20	0.9981
	40	15.80	0.05	0.9954	2.23	2.34	0.9848

Table S6. AC adsorption of DBP thermodynamic model parameters.

Ethanol volume fraction (v%)	T (K)	Thermodynamic equilibrium constant, K	ΔG (kJ/mol)	ΔH (kJ/mol)	ΔS (J/(mol K)
	298	3.9594	-3.3522		
30	303	4.1780	-3.6020	4.4681	26.67
	313	4.4524	-3.8864		
	298	2.7356	-2.4515		
50	303	2.8239	-2.6152	6.0591	28.90
	313	2.9441	-2.8100		
	298	0.2683	3.2049		
70	303	0.3034	3.0046	7.9159	16.12
	313	0.3301	2.8843		
	298	0.0953	5.7264		
100	303	0.0893	6.0856	35.441	99.94
	313	0.2444	3.6665		

Table S7. Physico-chemical properties of five PAEs concerned in this paper.

PAEs	M.W. (g/mol) [a]	Solubility in water at 25°C (g/L)[8]	Solubility parameter (MPa) $^{1/2}$[28]	Log K_{OW} [b]
DMP	194.2	<0.100	22.10	1.64
DEP	222.2	1.000	20.29	2.70
DBP	278.4	0.015	19.20	4.83
DEHP	390.6	<0.001	16.83	8.71
DnOP	390.6	<0.001	16.83	9.08

[a] Molecular weight. [b] Log K_{OW} (Chem Spider Database).

Table S8. Molecular size table of five PAEs concerned in this paper.

PAEs	Molecular Length (nm)[28]	Molecular Width (nm)[53]	Molecular Area (nm^2)	Molecular Volume(Å^3) [a]
DMP	1.048	0.366	0.384	178.43
DEP	1.190	0.405	0.482	221.79
DBP	1.315	0.406	0.593	298.96
DEHP	1.167	0.451	0.870	452.18
DnOP	1.451	0.409	0.775	458.74

[a] Obtained by molecular simulation software.

Table S9. Interaction energies of different simulated composite systems.

System	Interaction Energy (kcal/mol)					
	$E_{\text{Non-bond}}$		$E_{\text{van der Waals}}$		$E_{\text{electrostatic}}$	
	DBP-Solution	DBP-AC	DBP-Solution	DBP-AC	DBP-Solution	DBP-AC
DBP/ H_2O	-62.487	-120.077	-28.192	-110.842	-30.55	-6.73
DBP/30 v% Ethanol	-99.563	-56.587	-56.775	-50.363	-25.934	-3.704
DBP/50 v% Ethanol	-109.449	-29.121	-85.399	-23.858	-20.021	-3.616
DBP/70 v% Ethanol	-113.162	-18.881	-81.851	-15.121	-27.235	-1.351
DBP/100 v% Ethanol	-133.515	-24.313	-106.241	-20.463	-22.896	-1.38

Table S10. The position of the highest peak on both sides.

Ethanol contents (v%)	Distance (Å)		Height (Å)	
	Left	Right	Left	Right
0	4.7	5.8	3.7	3.0
50	3.8	7.7	3.1	2.6
100	9.7	11.2	1.7	1.2

Table S11. Adsorption capacity comparison of PAEs.

PAEs	Sample I[a] (mg/g)	Sample II[b] (mg/g)	Sample III[c] (mg/g)	Sample IV[d] (mg/g)
DMP			2.55	2.40
DEP			2.78	2.26
DBP	13.99	7.33	13.36	3.72
DEHP			17.52	8.48
DnOP			18.11	11.09

[a] Sample I is the solution of ethanol/water (50/50 v/v) added with DBP; [b] Sample II is the Baijiu II with DBP; [c] Sample III is the solution of ethanol/water (50/50 v/v) with five PAEs; [d] Sample IV is Baijiu II added with five PAEs.

Table S12. Adsorption capacity of AC for DBP in the manuscript.

Ethanol volume fraction (v%)	Conditiona (mg/g)	Conditionb (mg/g)	Conditionc (mg/g)	Conditiond (mg/g)	Conditione (mg/g)
0	16.39	16.39	-	-	-
30	9.70	15.47	161.29	163.93	149.25
50	7.01	13.99	78.13	81.97	64.10
70	3.05	7.89	36.23	51.02	51.02
100	1.58	2.34	21.23	19.65	15.80

[a]: The adsorption capacity at six hours. [b]: Equilibrium adsorption capacity. [c]: Equilibrium adsorption capacity calculated using Langmuir and Freundlich models at 20°C. [d]: Equilibrium adsorption capacity calculated using Langmuir and Freundlich models at 30°C. [e]: Equilibrium adsorption capacity calculated using Langmuir and Freundlich models at 40°C.

Table S13. Adsorption capacity of AC for multicomponent PAEs in the manuscript.

Ethanol volume fraction (v%)	Equilibrium adsorption capacity (mg/g)				
	DMP	DEP	DBP	DEHP	DnOP
0	22.23	21.5	18.21	17.49	18.28
50	2.55	2.78	13.36	17.52	18.11
100	1.38	0.56	0.41	4.60	10.10

Table S14. Adsorption capacity comparison of DBP in Baijiu.

Research result	Kind of wine	DBP initial concentration (mg/L)	Adsorption capacity (mg/g)
This paper	XiongMao Jiang-flavor style Baijiu	10.00	7.33
Tang[54]	RenHuai Jiang-flavor style Baijiu	5.38	2.45
Wang[55]	FengGu Strong-flavor style Baijiu	1.50	0.91
Dong[56]	RenHuai Strong-flavor style Baijiu	4.31	0.70
